# Efficient evolution of human antibodies from general protein language models

**DOI:** 10.1038/s41587-023-01763-2

**Published:** 2023-04-24

**Authors:** Brian L. Hie, Varun R. Shanker, Duo Xu, Theodora U. J. Bruun, Payton A. Weidenbacher, Shaogeng Tang, Wesley Wu, John E. Pak, Peter S. Kim

**Affiliations:** 1 Department of Biochemistry, Stanford University School of Medicine, Stanford, CA USA; 2 Sarafan ChEM-H, Stanford University, Stanford, CA USA; 3 Stanford Medical Scientist Training Program, Stanford University School of Medicine, Stanford, CA USA; 4 Department of Chemistry, Stanford University, Stanford, CA USA; 5 Chan Zuckerberg Biohub, San Francisco, CA USA

**Keywords:** Molecular evolution, Machine learning, Drug discovery

## Abstract

Natural evolution must explore a vast landscape of possible sequences for desirable yet rare mutations, suggesting that learning from natural evolutionary strategies could guide artificial evolution. Here we report that general protein language models can efficiently evolve human antibodies by suggesting mutations that are evolutionarily plausible, despite providing the model with no information about the target antigen, binding specificity or protein structure. We performed language-model-guided affinity maturation of seven antibodies, screening 20 or fewer variants of each antibody across only two rounds of laboratory evolution, and improved the binding affinities of four clinically relevant, highly mature antibodies up to sevenfold and three unmatured antibodies up to 160-fold, with many designs also demonstrating favorable thermostability and viral neutralization activity against Ebola and severe acute respiratory syndrome coronavirus 2 (SARS-CoV-2) pseudoviruses. The same models that improve antibody binding also guide efficient evolution across diverse protein families and selection pressures, including antibiotic resistance and enzyme activity, suggesting that these results generalize to many settings.

## Abstract (web summary)

A general protein language model guides protein evolution with 20 or fewer variants needed for testing.

## Main

Evolution searches across an immense space of possible sequences for rare mutations that improve fitness^1,2^. In nature, this search is based on simple processes of random mutation and recombination^1^, but using the same approach for directed evolution of proteins in the laboratory^3^ imposes a considerable experimental burden. Artificial evolution based on random guessing or brute force search typically devotes substantial effort to interrogate weakly active or non-functional proteins, requiring high experimental throughput to identify variants with improved fitness^4,5^.

**Fig. 1:**
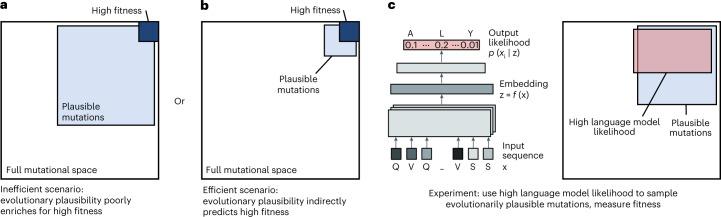
Guiding evolution with protein language models. **a**,**b**, Two possible models for relating the space of mutations with high evolutionary plausibility (for example, mutations seen in antibodies) to the space with high fitness under specific selection pressures (for example, mutations that result in high binding affinity to a specific antigen). Both models assume that mutations with high fitness make up a rare subset of the full mutational space and that, in general, high-fitness mutations are also evolutionarily plausible. Under the first model (**a**), mutations with high fitness are rare within the subset of mutations that are evolutionarily plausible. Under the second model (**b**), when restricted to the regime of plausible mutations, improvements to fitness become much more common. **c**, Protein language models, trained on millions of natural protein sequences learn amino acid patterns that are likely to be seen in nature. We hypothesized that most mutations with high language model likelihood would also be evolutionarily plausible. Assuming that this is true, and if the second model (**b**) better describes nature, then a language model with no information about specific selection pressures can still efficiently guide evolution.

Although evolutionary fitness is determined, in part, by specific selection pressures, there are also properties that apply more generally across a protein family or are prerequisites for fitness and function across most proteins; for example, some mutations maintain or improve stability or evolvability^6,7^, whereas others are structurally destabilizing^7^ or induce incompetent, misfolded states^8^. One approach to improving the efficiency of evolution is to ensure that mutations adhere to these general properties, which we refer to as evolutionary plausibility. Identifying plausible mutations could help guide evolution away from invalid regimes^9^, thereby indirectly improving evolutionary efficiency without requiring any explicit knowledge of the function of interest. However, this strategy is also challenging because, first, protein sequences are governed by complex rules, and, second, even if we restrict search to evolutionarily plausible mutations, those that also improve a specific definition of fitness might still be rare beyond practical utility (Fig. 1a). More broadly, a major open question^10^ is whether general evolutionary information (for example, learning patterns from sequence variation across past evolution) is sufficient to enable efficient evolution under specific selection pressures (for example, higher binding affinity to a specific antigen).

Here we show that evolutionary information alone can lead to improved fitness under specific selection pressures with high efficiency (Fig. 1b). For our main experimental test case, we focused on affinity maturation of human antibodies in which our specific selection pressure is defined as stronger binding affinity to a particular antigen. In nature, a process known as somatic hypermutation evolves or ‘matures’ an antibody lineage to have higher affinity for an antigen via repeated mutagenesis^11–13^. In the laboratory, affinity maturation is a major application of directed evolution due to the therapeutic potential of antibodies with high affinity for disease targets^14^.

To select evolutionarily plausible mutations, we used algorithms known as language models (Fig. 1c) to learn patterns that are likely to occur in natural proteins^15–22^. Because we used general language models^19,20^, trained on non-redundant sequence datasets that are meant to represent variation across all natural proteins^23^, these models can only learn more general evolutionary rules than could a model trained specifically on antibody sequences^24–27^ or a model directly supervised with binding affinity^28^. Given a single starting sequence, we used these language models to recommend plausible amino acid substitutions that we then experimentally screened for improved fitness. To the end user, the algorithm requires only a single wild-type sequence, without any initial binding affinity data, knowledge of the antigen, task-specific supervision, evolutionary homologs or protein structure information.

We evolved seven human immunoglobulin G (IgG) antibodies that bind to antigens from coronavirus, ebolavirus and influenza A virus. We focused on viral antigens given the importance of antibody therapeutics for viral diseases^29–32^. We improved the affinity of all antibodies after measuring only 20 or fewer new variants of each antibody across just two rounds of evolution, which, to our knowledge, represents unprecedented efficiency for machine-learning-guided evolution^33,34^. We also demonstrate that the *same* general protein language models that we used to affinity mature antibodies can also enrich for high-fitness substitutions to diverse proteins beyond antibodies.

## Results

### Efficient affinity maturation with protein language models

Recent work has demonstrated that language models can predict natural evolution despite having no knowledge of specific selection pressures^10^. However, this prior work only predicted the direction of evolution retrospectively when given full knowledge of the evolutionary trajectory. We hypothesized that the predictive capabilities of protein language models might enable a researcher to provide only a single, wild-type antibody sequence to the algorithm and receive a small, manageable set (~10^1^) of high-likelihood variants to experimentally measure for desirable properties. This is a very general setting that does not assume knowledge of protein structure or task-specific training data. A major question, however, is if higher evolutionary likelihood would efficiently translate to higher fitness.

We tested our hypothesis by conducting evolutionary campaigns, guided by language model likelihood, to affinity mature seven antibodies representing diverse antigens and degrees of maturity (Supplementary Table 1):

- MEDI8852: a broadly neutralizing antibody (bnAb) that binds influenza A hemagglutinin (HA) across variants of both major phylogenetic groups (group 1 and group 2) and that reached phase 2 clinical trials; this antibody is highly matured, with its parent being isolated from a human, followed by substantial artificial evolution^29^
- MEDI8852 unmutated common ancestor (UCA): the unmatured, inferred germline sequence of MEDI8852, which only neutralizes viruses with group 1 HAs^29^
- mAb114: a patient-derived antibody that neutralizes ebolavirus by binding to its glycoprotein (GP)^30^ and has been approved for clinical use by the US Food and Drug Administration (FDA)
- mAb114 UCA: the unmatured, inferred germline sequence of mAb114 with weak binding to ebolavirus GP^30^
- S309: a patient-derived antibody that cross-neutralizes the sarbecoviruses severe acute respiratory syndrome coronavirus 1 (SARS-CoV-1) and severe acute respiratory syndrome coronavirus 2 (SARS-CoV-2) by binding to the spike glycoprotein (Spike)^31^ and is the parent antibody of sotrovimab^35^, which had an FDA emergency use authorization (EUA) for treatment of Coronavirus Disease 2019 (COVID-19) caused by earlier variants of SARS-CoV-2 (refs. ^36,37^)
- REGN10987: a patient-derived antibody that binds early variants of SARS-CoV-2 Spike^32^ and that had an FDA EUA for use against these variants
- C143: an unmatured, patient-derived antibody that binds the SARS-CoV-2 Wuhan-Hu-1 Spike but was isolated before extensive in vivo somatic hypermutation^38,39^

We performed evolution with the ESM-1b language model and the ESM-1v ensemble of five language models (six language models in total)^19,20^. ESM-1b and ESM-1v were trained on UniRef50 and UniRef90, respectively, which are protein sequence datasets that represent variation across millions of observed natural proteins (UniRef90 contains ~98 million total sequences) and that include only a few thousand antibody-related sequences^23^. These datasets are also constructed such that no two sequences have more than 50% (UniRef50) or 90% (UniRef90) sequence similarity with each other to avoid biological redundancy. Additionally, both datasets precede the discovery of the SARS-CoV-2 antibodies considered in the study as well as the evolution of all SARS-CoV-2 variants of concern. Therefore, to evolve these antibodies, the language models cannot use disease-specific biases in the training data and must, instead, learn more general evolutionary patterns.

**Fig. 2:**
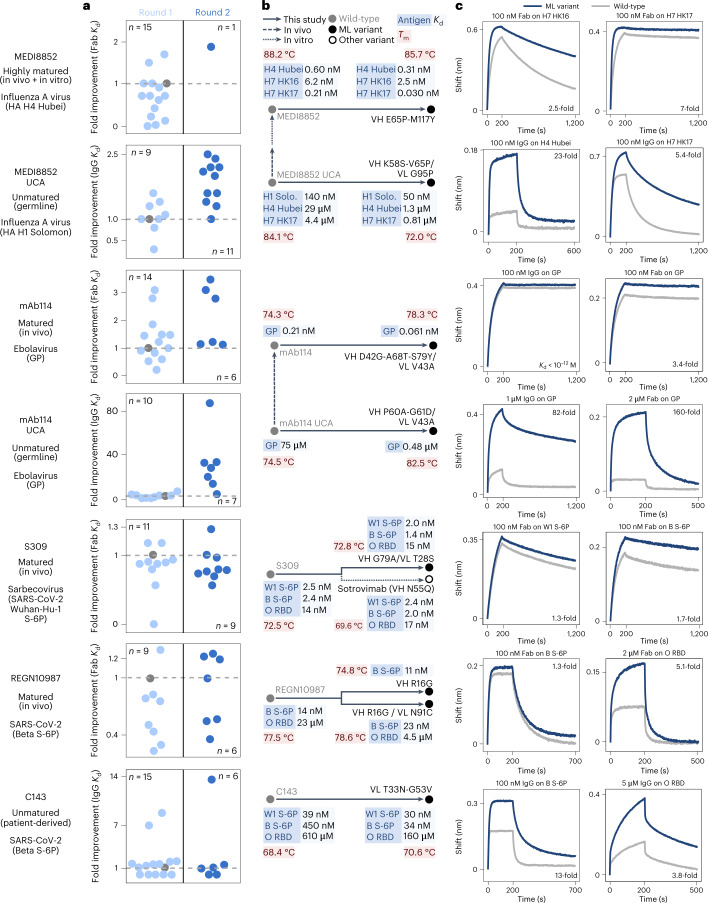
Language-model-guided affinity maturation of seven human antibodies. **a**, Strip plots visualizing the two rounds of directed evolution conducted for each antibody. Each point represents an IgG or Fab variant plotted according to the fold change in *K*_d_ from wild-type on the *y* axis and jitter on the *x* axis; a gray, dashed line is drawn at a fold change of 1, and the wild-type point is colored gray. MEDI8852 variants were screened against HA H4 Hubei; MEDI8852 UCA variants against HA H1 Solomon; mAb114 and mAb114 UCA variants against ebolavirus GP; S309 variants against Wuhan-Hu-1 S-6P; and REGN10987 and C143 variants against Beta S-6P. **b**, Phylogenetic trees illustrating the evolutionary trajectories from wild-type to the highest-affinity variant(s) of each antibody. Nodes are annotated with the *K*_d_ values for different antigens and the *T*_m_ of the Fab; all *K*_d_ values are for the monovalent Fab versions except those of C143, which are apparent *K*_d_ values for the bivalent IgGs. B, Beta; H1 Solo., H1 Solomon; ML variant, machine-learning-guided variant; O, Omicron; W1, Wuhan-Hu-1. **c**, We obtained avidity and affinity measurements via BLI of IgGs and Fabs at the indicated concentrations binding to the indicated antigen. Selected BLI traces of the highest-affinity variants for the respective antigens are plotted alongside those of the wild-type variants.

**Extended Data Fig. 1:**
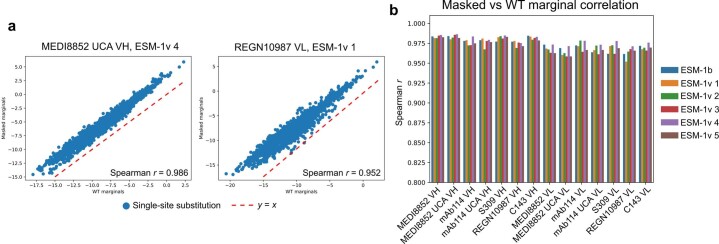
ESM masked versus wildtype marginals. (**a**) Representative scatter plots showing all possible single-site substitutions to an antibody sequence plotted according to their log-likelihood ratios to wildtype, where likelihoods are computed based on either masked marginals (*y-*axis) or wildtype marginals (*x*-axis). A red dashed line is plotted where masked and wildtype marginal values are equal. The wildtype marginal log-likelihoods are consistently lower overall, effectively serving to make the *α* parameter more stringent, while (**b**) the rank-based correlation between masked marginals and wildtype marginals is close to 1 in all cases.

We used these language models to compute likelihoods of all single-residue substitutions to the antibody variable regions of either the heavy chain (VH) or the light chain (VL). We selected substitutions with higher evolutionary likelihood than wild-type across a consensus of six language models (Methods and Extended Data Fig. 1). In the first round of evolution, we measured the antigen interaction strength by biolayer interferometry (BLI) of variants that contain only a single-residue substitution from wild-type. In the second round, we measured variants containing combinations of substitutions, where we selected substitutions that corresponded to preserved or improved binding based on the results of the first round. We performed these two rounds for all seven antibodies, measuring 8–14 variants per antibody in round one and 1–11 variants per antibody in round two (Fig. 2 and Supplementary Table 1). Variants of the clinically relevant antibodies, which have very low or undetectable dissociation as IgGs, were screened by measuring the dissociation constant (*K*_d_) of the monovalent fragment antigen-binding (Fab) region; variants of the unmatured antibodies were screened by measuring the apparent *K*_d_ of the bivalent IgG followed by also measuring the *K*_d_ values of the Fab fragments of the highest-avidity variants (Methods).

We could successfully express all but one of 122 variants across our seven evolutionary trajectories. Across all seven antibodies, we found that 71–100% of the first-round Fab variants (containing a single-residue substitution) retained sub-micromolar binding to the antigen, and 14–71% percent of first-round variants led to improved binding affinity (defined as a 1.1-fold or higher improvement in *K*_d_ compared to wild-type) (Supplementary Table 1). Most of the second-round variants (containing a combination of substitutions) also have improved binding (Supplementary Tables 1–9). For all antibodies except for REGN10987, we also obtained variants with at least a two-fold improvement in *K*_d_. Thirty-six out of all 76 language-model-recommended, single-residue substitutions (and 18 out of 32 substitutions that lead to improved affinity) occur in framework regions (Supplementary Tables 2–9), which are generally less mutated during conventional affinity maturation compared to the complementarity-determining regions (CDRs)^12^.

We were able to improve the binding affinities for all clinically relevant antibodies tested, despite these antibodies being already highly evolved (starting at low nanomolar or picomolar affinity). MEDI8852 is a potent binder with a sub-picomolar Fab *K*_d_ across many HAs and picomolar or nanomolar binding to HAs from subtypes H4 and H7. Although we explicitly screened variants using an HA H4 antigen, the best design also improves binding across a broad set of HAs (Supplementary Tables 2 and 3), including a sevenfold improvement (from 0.21 nM to 0.03 nM) for HA H7 HK17 (A/Hong Kong/125/2017(H7N9)). The best variant of mAb114, a clinically approved drug, achieves a 3.4-fold improvement in Fab *K*_d_ for ebolavirus GP (Supplementary Table 5). For REGN10987, the highest-affinity variant has a 1.3-fold improvement against Beta-variant Spike with six stabilizing proline substitutions (S-6P)^40^ (the antigen used in screening), and another of our designs has a 5.1-fold improvement for the Omicron BA.1 receptor-binding domain (RBD) (Supplementary Table 8). For S309, we compared our designs to wild-type and to a variant with the N55Q substitution in the VH introduced after a small-scale, rational evolutionary screen^35^; the S309 Fab with the VH N55Q substitution forms the Fab of the therapeutic antibody sotrovimab. Our best variant of S309 has higher affinity than sotrovimab, including a 1.3-fold improvement in Fab *K*_d_ compared to wild-type S309 (versus 1.1-fold for sotrovimab) for SARS-CoV-2 Wuhan-Hu-1 S-6P (the antigen used in screening); a 1.7-fold improvement (versus 1.3-fold for sotrovimab) for Beta S-6P; and a 0.93-fold change (versus 0.82-fold for sotrovimab) for Omicron RBD (Supplementary Table 7).

We were also able to improve affinities for all three unmatured antibodies, often involving much higher fold changes than when evolving the matured antibodies, indicating easier evolvability with respect to affinity. For MEDI8852 UCA, the best Fab design achieves a 2.6-fold improvement in *K*_d_ against HA H1 Solomon (A/Solomon Islands/3/2006(H1N1)), the antigen used in screening. Our best designs also acquire breadth of binding to some group 2 HAs, including a 23-fold improvement for HA H4 Hubei (A/swine/Hubei/06/2009(H4N1)) and a 5.4-fold improvement for HA H7 HK17 (Supplementary Table 4). For mAb114 UCA, our best Fab design achieves a 160-fold improvement in *K*_d_ for ebolavirus GP (Supplementary Table 6). Although the algorithm recommends amino acid substitutions to both of these UCA antibodies that are also observed in the matured antibody, other affinity-enhancing substitutions to the UCA antibodies are not found in the matured versions: excluding any substitutions or modified sites found in the matured antibody, our UCA variants achieve up to a sevenfold improvement for HA H4 Hubei (variant VH P75R/VL G95P; Supplementary Table 4) and a 33-fold improvement for ebolavirus GP (variant VH G88E/VL V43A; Supplementary Table 6), demonstrating that our algorithm successfully explores alternative evolutionary routes. For C143, a patient-derived antibody isolated before extensive affinity maturation^38,39^, our best design achieves a 13-fold improvement for Beta S-6P and a 3.8-fold improvement for Omicron RBD (Supplementary Table 9). Results from our directed evolution campaigns are further summarized in Fig. 2, Supplementary Tables 2–9 and Supplementary Data 1.

### Additional characterization of evolved antibodies

Although we explicitly selected for improved binders, we also tested these variants for improved stability (Methods). We found that Fabs for 21 out of the 31 language-model-recommended, affinity-enhancing variants that we tested had a higher melting temperature (*T*_m_) than wild-type, and all variants maintained thermostability (*T*_m_ > 70 °C). When evolving S309 to have higher affinity, our best design has a *T*_m_ of 72.8 °C compared to 72.5 °C for wild-type, whereas the VH N55Q substitution introduced in sotrovimab decreases the *T*_m_ to 69.6 °C (Fig. 2). Our evolved variants for mAb114, mAb114 UCA, REGN10987 and C143 also preserve or improve *T*_m_; the highest change that we observed was an increase from 74.5 °C to 82.5 °C when evolving mAb114 UCA. Improved thermostability does not completely explain our affinity maturation results, however, as we observed somewhat decreased *T*_m_ for our affinity-matured variants of MEDI8852 and its UCA, although these Fabs are still thermostable (Fig. 2).

**Fig. 3:**
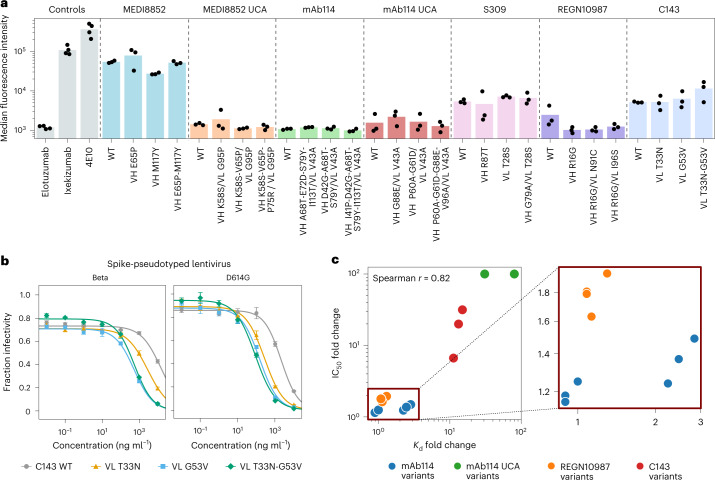
Specificity and improved neutralization potency of affinity-matured variants. **a**, Polyspecificity of antibody wild-types and variants was quantified using an assay^42^ that measures non-specific binding to soluble membrane proteins via flow cytometry, where higher MFI values correspond to more non-specific binding (Methods). Control antibodies^42^ are elotuzumab (a clinical antibody with low polyspecificity), ixekizumab (a clinical antibody with high polyspecificity) and 4E10 (a research antibody with high polyspecificity beyond a therapeutically viable level)^62^. Bar height indicates the mean across *n* = 3 replicate wells; black dots indicate independent measurements. **b**, Variants of the antibody C143, obtained from our language-model-guided affinity maturation campaign, demonstrate improved neutralization activity in a pseudovirus assay. For Beta pseudovirus, out of the three higher-affinity variants that we also screened for neutralization activity, the best improvement is the 32-fold improvement of VL G53V; for D614G pseudovirus, the best improvement is the 19-fold improvement of VL T33N-G53V (Supplementary Table 9). Also see Extended Data Fig. 2. Points indicate the mean; error bars indicate the s.d.; *n* = 4 independent experiments. **c**, Fold change in *K*_d_ correlates well with fold change in IC_50_ (Spearman *r* = 0.82, *n* = 15 antibody variants) across all designs tested, consistent with higher binding affinity contributing to improved viral neutralization activity. WT, wild-type.

Additionally, we tested our affinity-matured designs for polyspecific binding, because binding unintended targets could lead to undesirable side effects in therapeutic settings. For each of the seven antibodies, we tested the wild-type alongside three affinity-matured variants using a polyspecificity assay that assesses non-specific binding to soluble membrane proteins (Methods)^41,42^. We observed no substantial changes in polyspecificity for any variants of all seven antibodies, and all tested antibodies have polyspecificity values within a therapeutically viable range (Fig. 3a and Supplementary Data 2).

Another therapeutic consideration is immunogenicity. Although computational prediction of immunogenicity remains a challenge, especially involving recognition of discontinuous epitopes, the immunogenicity of linear peptides is better understood^43^. We observed that our affinity-matured variants have no significant increase (one-sided binomial *P* > 0.05) in the number of computationally predicted peptide binders to both human leukocyte antigen (HLA) class I and class II (exact *P* values and sample sizes for these experiments are provided in Supplementary Data 2), which underlies T-cell-mediated immunogenicity.

**Extended Data Fig. 2:**
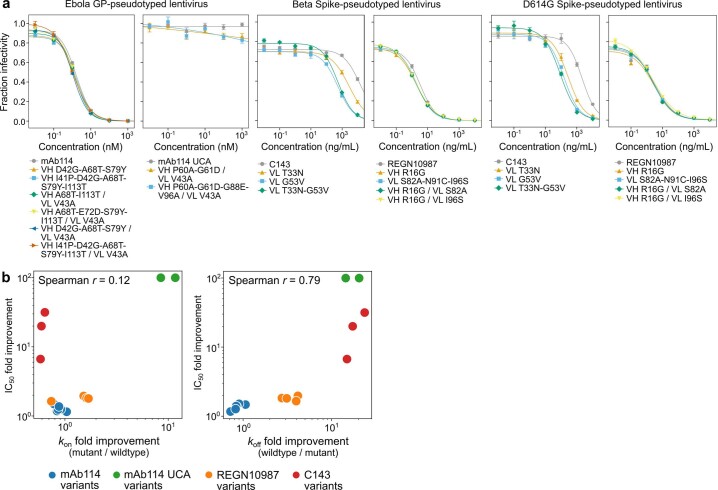
Pseudovirus neutralization of affinity-matured variants. (**a**) Neutralization curves for wildtype antibodies (gray) and variants obtained by our language-model-guided affinity maturation campaigns. Also see Supplementary Tables 5, 8, and 9 for corresponding IC_50_ values. Points indicate the mean; error bars indicate the standard deviation; *n* = 4 independent assays. (**b**) Fold-improvement in *k*_on_ has low correlation with fold-change in IC_50_ (Spearman *r* = 0.12), while fold-improvement in *k*_off_ has high correlation with fold-change in IC_50_ (Spearman *r* = 0.79); compare to Fig. 3c. Correlations involve *n* = 15 antibody variants. We define a higher *k*_on_ and a lower *k*_off_ as improved, so we divide the mutant value by the wildtype value to calculate fold-improvement in *k*_on_ and vice-versa to calculate fold-improvement in *k*_off_.

We also wanted to determine if our affinity-matured variants have better viral neutralization activity. We tested affinity-enhancing variants of four antibodies using pseudovirus neutralization assays (Methods) and, in all cases, observed variants with half-maximal inhibitory concentration (IC_50_) values that are significantly improved (Bonferroni-corrected, one-sided *t*-test *P* < 0.05, *n* = 4 independent experiments), including a 1.5-fold improvement for the best mAb114 variant against Ebola pseudovirus; a twofold improvement for the best REGN10987 variant against SARS-CoV-2 Beta pseudovirus; and a 32-fold improvement for the best C143 variant against Beta pseudovirus (Fig. 3b, Extended Data Fig. 2 and Supplementary Tables 5, 8 and 9). Additionally, the affinity-matured variants of mAb114 UCA demonstrate detectable neutralization at a >100-fold lower concentration compared to wild-type (Extended Data Fig. 2a). In general, change in binding affinity corelates well with change in neutralization (Spearman *r* = 0.82, two-sided *t*-distribution *P* = 1.9 × 10^−4^, *n* = 15 antibody variants) (Fig. 3c and Extended Data Fig. 2b).

### Originality of affinity-enhancing substitutions

Although the ability to find any improvement in affinity is itself useful for engineering applications, we were also interested in whether some of the changes recommended by our algorithm demonstrate ‘originality’. We quantified originality by computing the frequency that a given residue is observed in nature (Methods). Although many affinity-enhancing substitutions are indeed observed at high frequency both in the model’s training data^23^ and in a database of antibody sequences^44^, other substitutions demonstrate greater originality. For example, in the MEDI8852 UCA trajectory, the VL G95P framework substitution (Fig. 2 and Supplementary Table 4) involves changing a glycine observed in 99% of natural antibody sequences to a proline observed in less than 1% of natural sequences. Overall, five out of 32 affinity-enhancing substitutions (~16%) involve changing the wild-type residue to a rare or uncommon residue (Supplementary Table 10) and that are also rare when considering only natural variation of antibodies derived from the same germline genes (Supplementary Table 11). These results indicate that the language models learn both the ‘easy’ evolutionary rules involving high-frequency residues and more complex rules that are not captured by a multiple sequence alignment or conventional antibody evolution. Conceptually, these low-frequency, affinity-enhancing substitutions are analogous to examples in other disciplines where an artificial intelligence program occasionally makes unusual but advantageous choices (for example, unintuitive game-playing decisions^45^) and likewise may be worth further study.

### Comparison to other sequence-based methods

We also sought to compare general language models to other methods for selecting plausible mutations based on sequence information alone. To assess the contribution of epistatic information learned by the language model, we considered two site-independent models of mutational frequencies: (1) abYsis sequence annotation, which uses extensively curated antibody sequence alignments, and (2) frequencies based on sequence alignments to the UniRef90 dataset, which was used to train ESM-1v (Methods). To assess the impact of using language models not trained on antibody-specific sequence variation, we also compared to two antibody language models: (1) AbLang^24^, trained on ~10^7^ sampled sequences from immune repertoire sequencing data in the Observed Antibody Space (OAS) database^46^, and (2) Sapiens^25^, trained on ~10^8^ human antibody sequences from the OAS datasbase.

We benchmarked these models based on their ability to suggest single-residue substitutions that improve the avidity of the three unmatured IgG antibodies for their respective antigens (MEDI8852 UCA and HA H1 Solomon, mAb114 UCA and GP and C143 and Beta S-6P). For each of the four benchmarked models, we ranked substitutions by their mutant-to-wild-type likelihood ratios and experimentally tested the same number of substitutions considered in the first round of our evolutionary campaigns (Methods).

**Extended Data Fig. 3:**
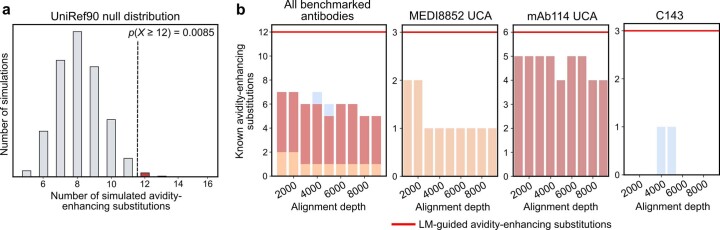
UniRef90 significance and robustness analysis. (**a**) A histogram of the null distribution generated by simulating how many avidity-enhancing substitutions would be recommended from a site-independent model based on UniRef90 alignments. Results are for *n* = 4.5 million simulations as described in Methods. Based on this null distribution and given that the language models recommended 12 avidity-enhancing substitutions, we estimate *P* = 0.0085. (**b**) The number of known avidity-enhancing substitutions recommended by a UniRef90 site-independent model at varying alignment depths, where our benchmark analyses are performed using an alignment depth of 10,000. The red line indicates the number of avidity-enhancing substitutions found by the language models. The combined number of known avidity-enhancing substitutions is provided in the stacked bar plot on the left and are separated by the antibody in the three right panels. The substitutions corresponding to each alignment depth and antibody are provided in Supplementary Data 3.

Notably, our approach based on general protein language models consistently outperformed all baseline methods (Supplementary Table 12). In particular, the abYsis and UniRef90 comparisons indicate that epistatic information was critical for consistent performance across antibodies. For example, the site-independent models did not recommend high-fitness substitutions such as VL G95P in MEDI8852 UCA or VL T33N/G53V in C143, resulting in no avidity-enhancing substitutions to C143 (Supplementary Table 12 and Supplementary Data 3). We also observed that language models recommend a significantly higher number of avidity-enhancing substitutions (simulation-based *P* = 0.0085; Extended Data Fig. 3a) compared to the next-best baseline, UniRef90, and that is robust to differences in sequence alignment depth (Extended Data Fig. 3b, Supplementary Data 3 and Methods). Despite having access to antibody-specific sequence variation, both the AbLang and Sapiens models also consistently underperformed the general protein language models and even underperformed the site-independent models when recommending substitutions to mAb114 UCA (Supplementary Table 12 and Supplementary Data 3). Our results indicate that general protein language models go beyond site-independent reasoning to make beneficial predictions while also learning sufficient information even from unspecialized protein sequence corpuses.

### Computational efficiency of our approach

Our computational pipeline is highly efficient at making predictions, taking less than 1 s per antibody (including both VH and VL sequences) on widely available, GPU-accelerated hardware (Methods). To demonstrate efficiency, we made predictions over 742 therapeutically relevant antibodies from the Thera-SAbDab database^47^ (Supplementary Data 4) in ~3 min, and our approach scales linearly with the number of antibodies.

### Generality across diverse protein families

Given the success of general protein language models at guiding antibody evolution, we also tested how well the same models could acquire high-fitness variants across diverse protein families. Previous work has demonstrated that the likelihoods from general protein language models have good correlation with experimental phenotypes from high-throughput assays over ~10^3^ to 10^4^ variants^10,20^. Previous computational simulations have also indicated that these models can help bias multi-round evolution away from large regions of a sequence landscape with zero or very low fitness^9^.

Here, we observed that the same models can also guide efficient evolution when measuring only a small number (~10^1^) of variants according to diverse definitions of fitness, including antibiotic resistance, cancer drug resistance, enzyme activity or viral replication fitness^48^. We used the same algorithm and language models in our affinity maturation experiments to suggest a small number (~10^1^) of changes to wild-type sequences from human, bacterial or viral organisms representing eight diverse protein families. We then used experimental measurements from high-throughput scanning mutagenesis experiments^8,48^ to validate the language-model-recommended predictions (notably, these measurements were not provided to the model). As in the antibody evolution campaigns, we are interested in enriching for as many high-fitness variants as possible among the small number of language model recommendations (rather than predicting fitness across the entire mutational space, as previously done^20^).

**Fig. 4:**
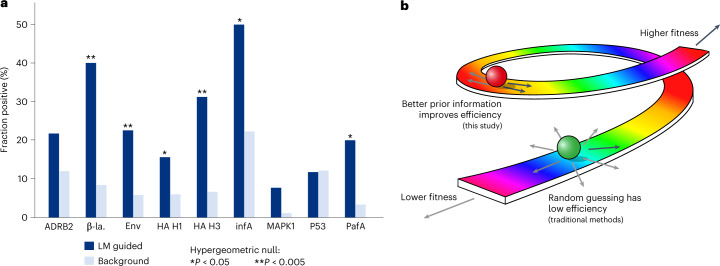
Guiding evolution without explicitly modeling fitness. **a**, The same strategy and language models that we use to affinity mature antibodies can also recommend high-fitness changes across a diversity of selection pressures and protein families, as identified experimentally using high-throughput scanning mutagenesis assays^8,48^ (described in Supplementary Table 13). ‘Fraction positive’ indicates the percentage of high-fitness amino acid substitutions within either the set of substitutions recommended by the language model (LM guided) or the set of all single-residue substitutions (Background). A large portion of language-model-guided substitutions have high fitness, which, in many cases, is significantly enriched compared to the background percentage; also see Extended Data Figs. 4–6, and see Supplementary Table 13 for the exact one-sided hypergeometric *P* values and sample sizes. ADRB2, adrenoreceptor beta 2; β-la., β-lactamase; Env, envelope glycoprotein; infA, translation initiation factor 1; MAPK1, mitogen-activated protein kinase 1; PafA, phosphate-irrepressible alkaline phosphatase. **b**, Conceptually, the prior information encoded by evolutionary plausibility is represented in this cartoon by the rainbow road, where ascending corresponds to improving fitness and descending corresponds to lowering fitness. Moving in any direction (for example, via random or brute force mutagenesis) would most likely decrease fitness or have a high chance of being a detrimental change (represented by the green ball). However, if evolutionary plausibility is an efficient prior (Fig. 1b), then movement that is constrained to the plausible regime (for example, when guided by a language model) substantially increases the chance of improving fitness (represented by the red ball).

**Extended Data Fig. 4:**
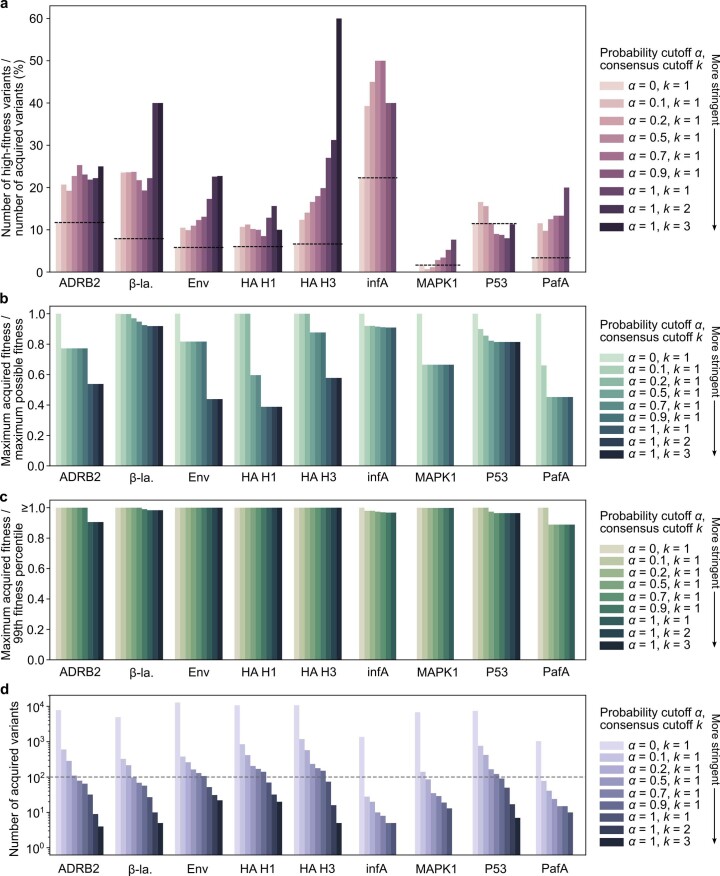
Relationship between likelihood stringency and fitness efficiency. To obtain the set ${{{\mathcal{A}}}}$ of language-model-recommended variants, we varied two parameters controlling the stringency of acquired variants (where more stringent corresponds to fewer variants): *α* is a cutoff controlling the likelihood ratio of the mutant probability to the wildtype probability, and *k* is a cutoff controlling the number of consensus language models (Methods). (**a**) At varying cutoffs, we computed the percentage fraction of variants in ${{{\mathcal{A}}}}$ that correspond to high-fitness variants, using scanning mutagenesis data for validation. When *α* = 0 and *k* = 1, this value is equivalent to the percentage of high-fitness variants in the full scanning mutagenesis dataset (a black dashed line is also drawn at this value for each protein). In all cases except for P53, we observe that increasing the likelihood stringency generally improves the efficiency at which high-fitness variants are acquired. In Fig. 4, we report values for *α* = 1, *k* = 2, except for when these cutoffs result in $\left| {{{\mathcal{A}}}} \right|$ < 5 (infA, MAPK1, and PafA), in which case we report *α* = 1, *k* = 1. (**b, c**) Given a set of acquired variants ${{{\mathcal{A}}}}$ at varying cutoffs, we also computed how much the maximum fitness represented in ${{{\mathcal{A}}}}$ compares either to the maximum possible fitness value obtained across the full mutational scan (**b**) or to the 99^th^ percentile of fitness values across the full mutational scan (**c**). To compare across proteins, we plotted the maximum acquired fitness value normalized by the maximum possible fitness (**b**) or by the 99^th^ percentile with a threshold at 1 (**c**). At even at the most stringent cutoffs, the best acquired variant of most proteins has at least 50% of the fitness value of the maximum fitness peak. Additionally, at the most stringent cutoffs, the best acquired variant of all proteins is above or close to the 99^th^ percentile of fitness values. (**d**) We plotted the number of acquired variants $\left| {{{\mathcal{A}}}} \right|$, which is the denominator of the values plotted in (**a**). A gray horizontal dashed line is also plotted at 100.

**Extended Data Fig. 5:**
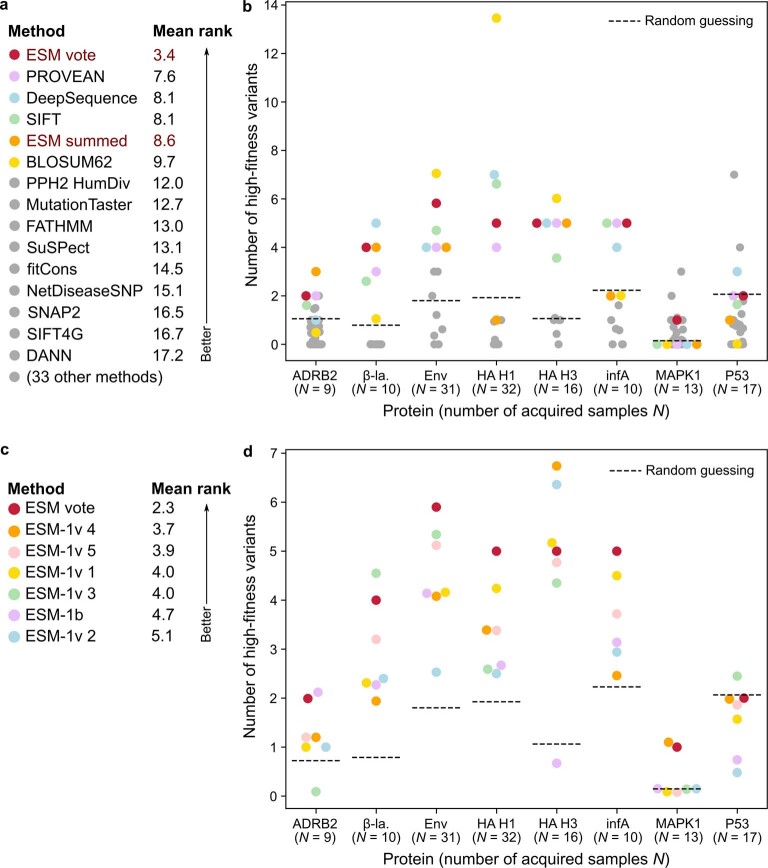
Benchmarking enrichment of high-fitness variants. (**a, b**) Variant effect prediction methods were ranked by the number of high-fitness variants acquired, controlling for the sample size *N* of total acquired variants used in Fig. 4, and ordered by the mean rank across eight proteins (Methods). Our consensus voting strategy (‘ESM vote’) ranks higher on average than all other methods based on its ability to acquire high-fitness variants. Methods profiled by Livesey and Marsh^48^ are in black text; ESM-based strategies profiled in this study are in red text. The full list of mean ranks is provided as Supplementary Data 5. ESM vote: the consensus strategy for acquiring substitutions used to select variants for experimental measurement in our antibody experiments. ESM summed: acquiring substitutions based on summed language model likelihood across the six language models used in this study. (**b**) Strip plot illustrating the number of high-fitness variants (vertical axis) among the top-*N* acquired substitutions to each protein (horizontal axis), where each point represents a different method for acquiring substitutions. These values are used to calculate the mean rank in (**a**). The expected number of variants that would be acquired via random guessing is plotted as a horizontal dashed line for each protein. (**c**, **d**) A similar analysis as in (**a**, **b**) but comparing the consensus voting strategy to each component of the ESM ensemble individually. Ensembling the recommendations across language models more consistently acquires high-fitness variants than when only using a single language model.

**Extended Data Fig. 6:**
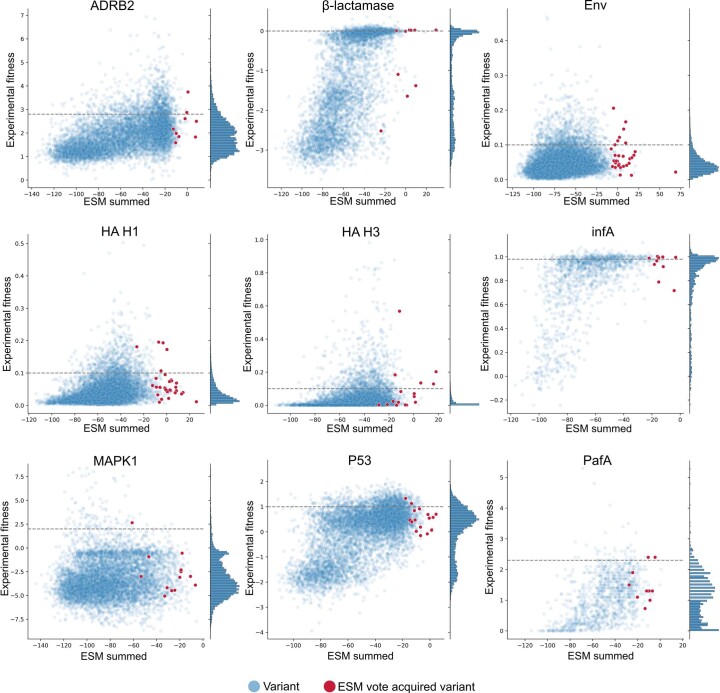
Scatter plots of DMS fitness data and ESM-ranked variants. Variants of each protein (with a single-site substitution from wildtype) are plotted as blue circles according to the experimentally-determined fitness value on the *y*-axis and the summed log-likelihood across the six ESM models considered in our analysis. The variants acquired by the ESM consensus voting scheme are plotted as red circles. The cutoff above which we define a high-fitness variant is plotted as a gray dashed line. The marginal distribution of experimental fitness values is also plotted as a histogram along the *y*-axis.

Language-model-recommended variants were nominally enriched (one-sided hypergeometric *P* < 0.05; exact *P* values and sample sizes are provided in Supplementary Table 13) for high-fitness values in six out of nine of the measured datasets, and high-fitness variants made up a much larger portion of language-model-recommended variants compared to random guessing in all but one case (Fig. 4a, Extended Data Figs. 4–6 and Supplementary Table 13). For example, whereas high ampicillin resistance is observed for just 7% of all single-residue substitutions to β-lactamase, it is observed for 40% of language-model-recommended substitutions, and the same set of language models can also help prioritize single-residue substitutions to HA that result in high viral infectivity (from 7% to 31%) and substitutions to PafA that improve enzyme kinetics (from 3% to 20%). Additionally, across all proteins, even the first round of a small-scale evolutionary campaign guided by language models would yield variants that are above or near the 99th percentile of fitness values (Extended Data Fig. 4). Compared to 47 alternative variant effect predictors, including supervised and structure-based models, our strategy ranks higher, on average, than all other methods based on the ability to recommend high-fitness variants (Extended Data Fig. 4, Supplementary Data 5 and Methods).

## Discussion

**Extended Data Fig. 7:**
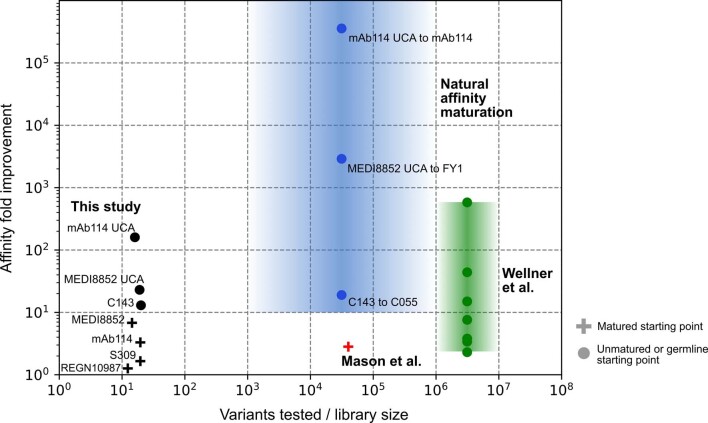
Comparison of affinity fold improvements versus experimental scale. Points indicate the results of affinity maturation beginning with an unmatured starting point (indicated by circles) or with a matured starting point (indicated by plus signs). The horizontal axis indicates the experimental scale in terms of variants tested or the experimental library size. The vertical axis indicates the fold improvement obtained by affinity maturation. Results from this study are plotted in black. While there is substantial uncertainty about the size of the mutational space explored by in-vivo somatic hypermutation (to include the unproductive B cell clones), we estimate a scale between 10^3^ to 10^6^ based on the number of B cells contained within a germinal center (about 10^3^ to 10^4^)^76,77^, the mutation rate of somatic hypermutation (about 1 mutation per kb per division)^13^, the doubling time of B cells (about 10 hours)^76^, and a timescale of a few weeks^13^. The results of natural affinity maturation of the unmatured antibodies in this study^29,30,38^, are plotted as blue dots (Supplementary Data 1). We also plot the results of recent studies reporting advances in antibody engineering technologies, including Mason et al.^28^ who achieve a 3-fold improvement in the binding of trastuzumab to human epidermal growth factor receptor 2 (HER2) using a library of ~39 K variants and Wellner et al.^14^ who achieve between a 2.3- and 580-fold improvement in the binding of unmatured nanobodies to SARS-CoV-2 RBD (picked out of a naïve library) using a continuously evolving yeast system involving 10^6^ to 10^7^ sorted cells over four or more rounds of selection.

We show that general protein language models can guide highly efficient affinity maturation based on the wild-type antibody sequence alone. Although our affinity improvements are lower than those typically observed in successful in vivo evolutionary trajectories, somatic hypermutation explores a mutational space that is larger by multiple orders of magnitude (Extended Data Fig. 7). Moreover, our affinity improvements on unmatured antibodies are within the 2.3-fold to 580-fold range previously achieved by a state-of-the-art, in vitro evolutionary system applied to unmatured, anti-RBD nanobodies (in which the computational portion of our approach, which takes seconds, is replaced with rounds of cell culture and sorting, which take weeks)^14^ (Extended Data Fig. 7). In vitro, cell surface display methods also encounter physical limits that make it challenging to distinguish better binders when the wildtype binder already has high affinity (<1 nM)^5^, which is not a limitation of our approach.

More broadly, a critical finding of our study is that evolutionary information alone provides sufficient prior information when selecting small numbers of substitutions to test for improved fitness (Figs. 1b and 4b). This leads to the result that a model without any task-specific training data or knowledge of the antigen can guide antibody evolution toward higher binding affinity, with competitive performance compared to protein-specific or task-specific methods (Supplementary Table 12 and Extended Data Fig. 5). We hypothesize that, in many settings, when mutations are constrained to follow a set of general evolutionary rules, a substantial portion (greater than 10%) is bound to improve fitness (Fig. 4b), which has immediate and broader implications for evolution in the laboratory and in nature.

### Practical implications and extensions

We anticipate that language models will become a key part of the antibody engineer’s toolkit, particularly within preclinical development as a rapid way to identify improved variants. In addition to speed, by focusing on ~10 single-site substitutions, a higher-throughput experimental budget that would have been allocated to brute force search could, instead, be allocated to exploring combinations of mutations^49,50^ or to exploring variants of more wild-type antibodies. Language-model-guided evolution could also complement or replace random mutagenesis strategies based on, for example, an error-prone polymerase.

To the end user, guiding evolution via pre-trained, unsupervised models is less resource intensive than collecting enough task-specific data to train a supervised model^28^. Language models should also serve as a baseline for future machine learning methods using supervision or other task-specific training data. Our techniques can also be used in conjunction with supervised approaches^9,28,33,34,51–54^, and supervising a model over multiple experimental rounds might ultimately lead to higher fitness. However, in many practical settings (for example, the rapid development of sotrovimab in response to the COVID-19 pandemic^35^), the efficiency of an unsupervised, single-round approach is preferable to a protracted, multi-round directed evolution campaign.

A general approach not biased by traditional structural hypotheses can also be valuable because many beneficial mutations are structurally remote to functionally important sites^55^. About half of the language-model-recommended substitutions (and about half of the affinity-enhancing substitutions) fall in framework regions, which are typically not proximal to the binding interface and are, therefore, sometimes excluded from directed evolution^28,56^. Although some of these framework changes may improve affinity via protein stabilization, others do not appear to increase thermostability (for example, VL G95P in MEDI8852 UCA) and may, instead, be causing affinity improvements via structural reorientation^57–59^. Nature often takes advantage of framework mutations to improve affinity, which represent ~20–30% of changes in natural affinity maturation^60^. In one well-known case, none of the nine residues accounting for a 30,000-fold increase in affinity is in contact with the antigen^59^, and, in another case, framework mutations make important contributions to affinity maturation and increased breadth in an HIV-1 antibody^58^.

### Generality of fitness improvements

By leveraging general evolutionary rules, language models recommend more ‘universal’ changes that seem to generalize better when the definition of fitness changes (Fig. 4). We also observed that general language models outperform antibody-specific language models (Supplementary Table 12), which is consistent with independent in silico benchmarking^22^. When transferring to a new, specific notion of fitness, more general evolutionary information may outweigh the particular biases encoded in antibody repertoire datasets, although further development of antibody language models could improve performance.

Our general approach is designed to improve an existing baseline function (for example, improving the affinity of a weak binder) rather than endowing any protein with an arbitrary function (for example, converting a generic protein into a specific binder). We also note that taking advantage of this strategy for guiding evolution may be more difficult when the selection pressure is unnatural or if the wild-type sequence is already at a fitness peak. However, in many practical design tasks, natural sequences and selection pressures are already preferrable; for example, therapeutic development often prefers human antibodies due to considerations of immunogenicity.

Beyond protein engineering, the success of our approach may also provide insight into natural evolution. The efficiency of evolutionary information alone may reflect natural mechanisms for biasing mutation rates toward higher fitness: for example, somatic hypermutation favors specific parts of an antibody gene via epigenomic and enzymatic sequence biases^60,61^. If epigenomic or other mechanisms predispose mutations to have high fitness, then nature could be accelerating evolution in a manner similar to our approach.

## Methods

### Acquiring amino acid substitutions via language model consensus

We select amino acid substitutions recommended by a consensus of language models. We take as input a single wild-type sequence *x* = (*x*_1_,…,*x*_*N*_)∈ $\mathcal{X}$^*N*^, where $\mathcal{X}$ is the set of amino acids, and *N* is the sequence length. We also require a set of masked language models, which are pre-trained to produce conditional likelihoods $p\left( {x_i^\prime |{{{\mathbf{x}}}}} \right)$. To guide evolution based on a certain language model, we first compute the set of substitutions with higher language model likelihood than the wild-type—that is, we compute the set

$${{{\mathcal{M}}}}\left( {p_j} \right) = \left\{ {i \in \left[ N \right],x_i^\prime \in {{{\mathcal{X}}}}:\frac{{p_j\left( {x_i^\prime |{{{\mathbf{x}}}}} \right)}}{{p_j\left( {x_i|{{{\mathbf{x}}}}} \right)}} > \alpha } \right\},$$

where *p*_*j*_ denotes the language model, *x*_*i*_ denotes the wild-type residue and *α* = 1. To further filter substitutions to only those with the highest likelihood, we choose substitutions based on a consensus scheme, where, for a new amino acid $x_i^\prime$, we compute

$$f\left( {x_i^\prime } \right) = \mathop {\sum}\limits_{j \in \left[ M \right]} 1 \left\{ {\left( {i,x_i^\prime } \right){{{\mathrm{is}}}}\,{{{\mathrm{in}}}\,}{{{\mathcal{M}}}}\left( {p_j} \right)} \right\}$$

where 1{·} denotes the indicator function, and there are *M* language models. We then acquire the set of substitutions with higher likelihood than wild-type across multiple language models—that is, we acquire

$${{{\mathcal{A}}}} = \left\{ {i \in \left[ N \right],x_i^\prime \in {{{\mathcal{X}}}}:f\left( {x_i^\prime } \right) \ge k} \right\}$$

where *k* is a user-supplied cutoff that controls the number of corresponding variants to measure. Although we focus on values of *k* that result in small values of $|{{{\mathcal{A}}}}|$ (around 10) that can be screened via low-throughput assays, the number of substitutions can be increased by reducing the value of *k* or by lowering the cutoff stringency *α*. Our strategy based on computing ‘wild-type marginal’ likelihoods based on the entire sequence, $p\left( {x_i^\prime |{{{\mathbf{x}}}}} \right)$, instead of the ‘masked marginal’ likelihoods in which the site of interest is masked, $p\left( {x_i^\prime |{{{\mathbf{x}}}}_{\left[ N \right]\backslash \left\{ i \right\}}} \right)$, also increases the cutoff stringency (Extended Data Fig. 1).

We use six large-scale masked language models—namely, the ESM-1b model^19^ and the five models that are ensembled together to form ESM-1v^20^—both obtained from https://github.com/facebookresearch/esm. ESM-1b was trained on the 2018-03 release of UniRef50 (ref. ^23^) consisting of ~27 million sequences, and the five models in ESM-1v were each trained on the 2020-03 release of UniRef90 (ref. ^23^) consisting of ~98 million sequences.

### Antibody sequence analysis and evolution

For antibodies, we performed the above steps for the VH and VL sequences separately, obtaining respective sets ${{{\mathcal{A}}}}_{{{{\mathrm{VH}}}}}$ and ${{{\mathcal{A}}}}_{{{{\mathrm{VL}}}}}$. For round 1 of evolution, we set *α* = 1 and chose values of *k* such that $|{{{\mathcal{A}}}}_{{{{\mathrm{VH}}}}} \cup {{{\mathcal{A}}}}_{{{{\mathrm{VL}}}}}|$ is approximately 10, which is meant to be a reasonable number of antibody variants for one person to express and purify in parallel. We used *k* = 2 for MEDI8852 VH and VL, *k* = 2 for MEDI8852 UCA VH and VL, *k* = 4 for mAb114 VH and VL, *k* = 2 for mAb114 UCA VH and VL, *k* = 2 for S309 VH, *k* = 1 for S309 VL, *k* = 2 for REGN10987 VH and VL and *k* = 2 for C143 VH and VL. We further reduced the size of $|{{{\mathcal{A}}}}_{{{{\mathrm{VH}}}}} \cup {{{\mathcal{A}}}}_{{{{\mathrm{VL}}}}}|$ by requiring the substitution to have the highest likelihood at its respective site for at least one language model. Variants were first measured for binding affinity to a given antigen via BLI (more details below), and those that enhanced affinity were recombined such that the second-round variants have two or more substitutions from wild-type, which were tested during round 2 of evolution. Given the small number of affinity-enhancing substitutions found during round 1 of evolution for S309 and REGN10987, we also expanded the set of substitutions considered in round 2 to include those that preserved affinity. For MEDI8852 and MEDI8852 UCA, we tested all possible combinations in round 2; for the other antibodies, where the number of possible combinations far exceeds ~10 variants, we manually selected a set of combinations meant to prioritize inclusion of substitutions that resulted in the largest improvements in affinity during the first round.

We used the wild-type sequences provided by the original study authors describing the respective antibodies^29–32,38^. Wild-type VH and VL sequences are provided in the Supplementary Information. We used the Kabat region definition provided by the abYsis webtool version 3.4.1 (http://www.abysis.org/abysis/index.html)^44^ to annotate the framework regions and CDRs within the VH and VL sequences.

### Antibody avidity benchmarking experiments

We also compared the substitutions recommended by the above strategy (based on language model consensus) to the substitutions recommended by four alternative sequence-based methods. First, we acquired substitutions to a VH or VL sequence based on site-independent mutational frequencies, where we used either the frequencies computed by the abYsis Annotation webtool^44^ or the frequencies obtained using all sequences in UniRef90 (the training dataset of ESM-1v)^23^. To compute the UniRef90 frequencies, we first performed an exhaustive search to obtain the 10,000 closest sequences by Levenshtein distance, where 10,000 is chosen to reflect the number of immunoglobulin-like sequences in UniRef90. We computed sequence similarity using the partial_ratio function from the FuzzyWuzzy Python package version 0.18.0; we then constructed a multiple sequence alignment of these 10,000 sequences using MAFFT version 7.475 (ref. ^63^) using the VH or VL sequence as the reference; finally, using the alignment, we computed mutational frequencies for each site in the sequence. We selected the top-ranking substitutions by likelihood ratio (the mutant frequency divided by the corresponding wild-type frequency) across the VH and VL sequences, where, for each antibody, we selected the same number of substitutions considered in the first round of our evolutionary campaigns.

We also acquired substitutions based on language models trained specifically on antibody sequences. We used the AbLang heavy chain and light chain language models (https://github.com/TobiasHeOl/AbLang)^24^ and the Sapiens heavy chain and light chain language models (https://github.com/Merck/Sapiens)^25^ to compute the mutant-to-wild-type likelihood ratios for all single-residue substitutions to the VH or VL sequence (using the language model trained on sequences from the corresponding chain). We selected the top-ranking substitutions by likelihood ratio across the VH and VL sequences and, following our use of the general protein language models, also required the substitution to have the highest likelihood at its site. For each antibody, we selected the same number of substitutions considered in the first round of our evolutionary campaigns.

We used these four methods (abYsis, UniRef90, AbLang and Sapiens) to select substitutions to our three unmatured antibodies (MEDI8852 UCA, mAb114 UCA and C143) and used BLI to measure IgG avidity to their respective antigens (HA H1 Solomon, GP and Beta S-6P). To purify the larger number of variants involved in these benchmarking studies, we used a medium-throughput system using a robotic liquid handler, described in more detail below. With this system, we expressed and purified antibody variants containing single-residue substitutions from wild-type recommended by the consensus of ESM language models as well as by the four baseline methods, observing similar purities and affinities when the same variants were also expressed and purified via the low-throughput system (described below) used in our evolutionary campaigns. Antibodies with a final concentration of less than 0.1 mg ml^−1^ in 200 μl after the medium-throughput purification were re-expressed and purified using the low-throughput methodology.

### UniRef90 robustness and statistical significance analysis

For the UniRef90 benchmark, we additionally assessed robustness to differences in multiple sequence alignment (MSA) construction by computing the number of known affinity-enhancing substitutions while varying the sequence alignment depth from 1,000 to 9,000 sequences at increments of 1,000 (for a total of nine alignment depth cutoffs). At each cutoff, we re-ran the procedure described above to select substitutions (constructing MSAs and calculating mutational likelihood ratios). We performed this for all three experimentally benchmarked antibodies, representing a total of 27 MSAs. Among the top-ranked substitutions for each cutoff and benchmarked antibody, we counted the number of known affinity-enhancing substitutions and provide the results in Extended Data Fig. 3 and Supplementary Data 3.

We also used the UniRef90 benchmark to assess the statistical significance of the number of avidity-enhancing substitutions recommended by the language models. In particular, we calculated the probability of acquiring 12 or more avidity-enhancing substitutions (Supplementary Table 12) by simulating different outcomes of a site-independent model based on UniRef90 alignments. To construct the null distribution, we first simulated variation in UniRef90 alignments using the nine MSAs of varying alignment depth and their corresponding recommended substitutions, described in the previous paragraph. We then simulated experimental measurement of these mutations for avidity enhancement across the three benchmarked antibodies: for each top-ranked substitution with an unknown effect on avidity, we assigned a success probability based on the observed probabilities from our experimental benchmark (2/8 = 25% for MEDI8852 UCA; 5/9 = 56% for mAb114 UCA; and 1/14 = 7% for C143); for each top-ranked substitution with a known effect on avidity, we fixed its value to its experimentally determined status. We ran 500,000 simulations for each of the nine MSA cutoffs (a total of 4.5 million simulations), where each simulation returns a total number of avidity-enhancing substitutions across the three antibodies. We report the *P* value as the number of simulations resulting in 12 or more avidity-enhancing substitutions divided by the total number of simulations.

### Antibody cloning

We cloned the antibody sequences into the CMV/R plasmid backbone for expression under a CMV promoter. The heavy chain or light chain sequence was cloned between the CMV promoter and the bGH poly(A) signal sequence of the CMV/R plasmid to facilitate improved protein expression. Variable regions were cloned into the human IgG1 backbone; REGN10987 and C143 variants were cloned with a lambda light chain, whereas variants of all other antibodies were cloned with a kappa light chain. The vector for both heavy and light chain sequences also contained the HVM06_Mouse (UniProt: P01750) Ig heavy chain V region 102 signal peptide (MGWSCIILFLVATATGVHS) to allow for protein secretion and purification from the supernatant. VH and VL segments were ordered as gene blocks from Integrated DNA Technologies and were cloned into linearized CMV/R backbones with 5× In-Fusion HD Enzyme Premix (Takara Bio); a list of oligonucleotides and gene blocks used in the study is provided as Supplementary Data 6.

### Antigen cloning

HA, GP, Spike and RBD sequences were cloned into a pADD2 vector between the rBeta-globin intron and β-globin poly(A). HA constructs contain a Foldon trimerization domain. GP and Spike constructs contain a GCN4 trimerization domain. All HAs, GP, Wuhan-Hu-1 S-6P and Omicron BA.1 RBD constructs contain an AviTag. All constructs contain a C-terminal 6×His tag. We used HA sequences from the following strains: A/New Caledonia/20/1999(H1N1) (H1 Caledonia), A/Solomon Islands/3/2006(H1N1) (H1 Solomon), A/Japan/305/1957 (H2N2) (H2 Japan), A/Panama/2007/1999(H3N2) (H3 Panama), A/Victoria/3/1975(H3N2) (H3 Victoria), A/swine/Hubei/06/2009(H4N1) (H4 Hubei), A/Vietnam/1203/2004(H5N1) (H5 Vietnam), A/Hong Kong/61/2016(H7N9) (H7 HK16) and A/Hong Kong/125/2017(H7N9) (H7 HK17). We used Ebola GP ectodomain (Mayinga, Zaire, 1976, GenBank: AAG40168.1) with the mucin-like domain deleted (Δ309–489). Spike or RBD sequences were based off wild-type Wuhan-Hu-1 (GenBank: BCN86353.1), Beta (GenBank: QUT64557.1) or Omicron BA.1 (GenBank: UFO69279.1).

### DNA preparation

Plasmids were transformed into Stellar competent cells (Takara Bio), and transformed cells were plated and grown at 37 °C overnight. Colonies were mini-prepped per the manufacturer’s recommendations (GeneJET, K0502, Thermo Fisher Scientific) and sequence confirmed (Sequetech) and then maxi-prepped per the manufacturer’s recommendations (NucleoBond Xtra Maxi, Macherey-Nagel). Plasmids were sterile filtered using a 0.22-μm syringe filter and stored at 4 °C.

### Protein expression

All proteins were expressed in Expi293F cells (Thermo Fisher Scientific, A14527). Proteins containing a biotinylation tag (AviTag) were also expressed in the presence of a BirA enzyme, resulting in spontaneous biotinylation during protein expression. Expi293F cells were cultured in media containing 66% FreeStyle/33% Expi media (Thermo Fisher Scientific) and grown in TriForest polycarbonate shaking flasks at 37 °C in 8% carbon dioxide. The day before transfection, cells were spun down and resuspended to a density of 3 × 10^6^ cells per milliliter in fresh media. The next day, cells were diluted and transfected at a density of approximately 3–4 × 10^6^ cells per milliliter. Transfection mixtures were made by adding the following components: maxi-prepped DNA, culture media and FectoPRO (Polyplus) would be added to cells to a ratio of 0.5 μg: 100 μl: 1.3 μl: 900 μl. For example, for a 100-ml transfection, 50 μg of DNA would be added to 10 ml of culture media, followed by the addition of 130 μl of FectoPRO. For antibodies, we divided the transfection DNA equally among heavy and light chains; in the previous example, 25 μg of heavy chain DNA and 25 μg of light chain DNA would be added to 10 ml of culture media. After mixing and a 10-min incubation, the example transfection cocktail would be added to 90 ml of cells. The cells were harvested 3–5 days after transfection by spinning the cultures at >7,000*g* for 15 min. Supernatants were filtered using a 0.45-μm filter.

### Antibody purification (low throughput)

We purified antibodies using a 5-ml MabSelect Sure PRISM column on the ÄKTA pure fast protein liquid chromatography (FPLC) instrument (Cytiva). The ÄKTA system was equilibrated with line A1 in 1× PBS, line A2 in 100 mM glycine pH 2.8, line B1 in 0.5 M sodium hydroxide, Buffer line in 1× PBS and Sample lines in water. The protocol washes the column with A1, followed by loading of the sample in the Sample line until air is detected in the air sensor of the sample pumps, followed by five column volume washes with A1, elution of the sample by flowing of 20 ml of A2 directly into a 50-ml conical containing 2 ml of 1 M tris(hydroxymethyl)aminomethane (Tris) pH 8.0, followed by five column volumes of A1, B1 and A1. We concentrated the eluted samples using 50-kDa or 100-kDa cutoff centrifugal concentrators, followed by buffer exchange using a PD-10 column (Sephadex) that had been pre-equilibrated into 1× PBS. Purified antibodies were stored at −20 °C.

### Antibody purification (medium throughput)

For our benchmarking experiments, we purified antibody variants with a medium-throughput system using an Agilent Bravo robotic liquid handling platform and VWorks software version 13.1.0.1366 with custom programming routines. For each antibody wild-type or variant, a 2.5-ml culture of Expi293F cells was transfected with corresponding antibody heavy and light chain plasmids as previously described. Cultures were harvested 3–5 days after transfection by centrifugation at 4,200*g* for 10 min, followed by collecting 2 ml of supernatant. ProPlus PhyTip column tips (Biotage, PTV-92-20-07) were loaded on the Bravo 96 LT head and equilibrated by aspirating and dispensing 75 μl of PBS, repeating four times. Sample binding to the tip resin was performed by aspirating and dispensing 98 μl of harvested supernatant, followed by washing via aspirating and dispensing 100 μl of PBS, repeating the binding and washing steps nine times (in total processing 882 μl of harvest for each run). Elution was performed by aspirating 100 μl of 100 mM glycine pH 2.8, followed by dispensing into a well with 10 μl of 1 M Tris pH 8.

### Antigen purification

All antigens were His-tagged and purified using HisPur Ni-NTA resin (Thermo Fisher Scientific, 88222). Cell supernatants were diluted with 1/3 volume of wash buffer (20 mM imidazole, 20 mM 4-(2-hydroxyethyl)-1-piperazineethanesulfonic acid (HEPES) pH 7.4, 150 mM sodium chloride (NaCl) or 20 mM imidazole, 1× PBS), and the Ni-NTA resin was added to diluted cell supernatants. For all antigens except SARS-CoV-2 Spike, the samples were then incubated at 4 °C while stirring overnight. SARS-CoV-2 Spike antigens were incubated at room temperature while stirring overnight. Resin/supernatant mixtures were added to chromatography columns for gravity flow purification. The resin in the column was washed with wash buffer (20 mM imidazole, 20 mM HEPES pH 7.4, 150 mM NaCl or 20 mM imidazole, 1× PBS), and the proteins were eluted with 250 mM imidazole, 20 mM HEPES pH 7.4, 150 mM NaCl or 20 mM imidazole, 1× PBS. Column elutions were concentrated using centrifugal concentrators at 10-kDa, 50-kDa or 100-kDa cutoffs, followed by size-exclusion chromatography on an ÄKTA pure system (Cytiva). ÄKTA pure FPLC with a Superdex 6 Increase (S6) or Superdex 200 Increase (S200) gel filtration column was used for purification. Then, 1 ml of sample was injected using a 2-ml loop and run over the S6 or S200, which had been pre-equilibrated in degassed 20 mM HEPES, 150 mM NaCl or 1× PBS before use and stored at −20 °C.

### Fab production and purification

Next, 1/10 volume of 1 M Tris pH 8 was added to IgGs at ~2 mg ml^−1^ in 1× PBS. Then, 2 μl of a 1 mg ml^−1^ stock of Lys-C (stock stored at −20 °C) was added for each milligram of human IgG1 and digested for 1 h at 37 °C with moderate rotation. Digested Fabs were purified using a 5-ml HiTrap SP HP cation exchange chromatography column on an ÄKTA system using 50 mM sodium acetate (NaOAc) pH 5.0 with gradient NaCl elution (using 50 mM NaOAc + 1 M NaCl pH 5.0). Fab fractions were pooled and dialyzed against 1× PBS and concentrated using 30-kDa concentrators. Purified Fabs were stored at −20 °C.

### BLI binding experiments

All reactions were run on an Octet RED96 at 30 °C, and samples were run in 1× PBS with 0.1% BSA and 0.05% Tween 20 (Octet buffer). IgGs and Fabs were assessed for binding to biotinylated antigens using streptavidin biosensors (Sartorius/ForteBio) or to unbiotinylated, His-tagged antigens using Anti-Penta-HIS biosensors (Sartorius/ForteBio). Antigen was loaded to a threshold of 1-nm shift. Tips were then washed and baselined in wells containing only Octet buffer. Samples were then associated in wells containing IgG or Fab at 100 nM concentration unless otherwise stated (other concentrations are given in Supplementary Data 1). A control well with loaded antigen but that was associated in a well containing only 200 μl of Octet buffer was used as a baseline subtraction for data analysis. Association and dissociation binding curves were fit in Octet System Data Analysis Software version 9.0.0.15 using a 1:2 bivalent model for IgGs to determine apparent *K*_d_ and a 1:1 model for Fabs to determine *K*_d_. Averages of fitted *K*_d_ values from at least two independent experiments are reported to two significant figures. Wild-type and the highest-affinity variants were also tested at multiple concentrations, and *K*_d_ values were averaged across all replicates and concentrations (Supplementary Data 1). To estimate measurement error, we computed the coefficient of variation (CV; the ratio of the s.d. to the mean across replicates) for each antibody−antigen *K*_d_ pair, and we report the mean CV for each antigen in Supplementary Tables 2 and 4–9.

### Thermal melts

We measured thermal melting profiles of proteins by differential scanning fluorimetry on a Prometheus NT.48 instrument. Protein samples (0.1 mg ml^−1^) were loaded into glass capillaries and then subjected to a temperature gradient from 20 °C to 95 °C at a heating rate of 1 °C per minute. Intrinsic fluorescence (350 nm and 330 nm) was recorded as a function of temperature using PR.ThermControl version 2.3.1 software. Thermal melting curves were plotted using the first derivative of the ratio (350 nm/330 nm). Melting temperatures were calculated automatically by the instrument and represented peaks in the thermal melting curves.

### PolySpecificity Particle assay

Polyspecificity reagent (PSR) was obtained as described by Xu et al.^41^. Soluble membrane proteins were isolated from homogenized and sonicated Expi 293F cells followed by biotinylation with Sulfo-NHC-SS-Biotin (Thermo Fisher Scientific, 21331) and stored in PBS at −80 °C. The PolySpecificity Particle (PSP) assay was performed following Makowski et al.^42^. Protein A magnetic beads (Invitrogen, 10001D) were washed three times in PBSB (PBS with 1 mg ml^−1^ BSA) and diluted to 54 μg ml^−1^ in PBSB. Then, 30 μl of the solution containing the beads was incubated with 85 μl of antibodies at 15 µg ml^−1^ overnight at 4 °C with rocking. The coated beads were then washed twice with PBSB using a magnetic plate stand (Invitrogen, 12027) and resuspended in PBSB. We then incubated 50 μl of 0.1 mg ml^−1^ PSR with the washed beads at 4 °C with rocking for 20 min. Beads were then washed with PBSB and incubated with 0.001× streptavidin-APC (BioLegend, 405207) and 0.001× goat anti-human Fab fragment FITC (Jackson ImmunoResearch, 109-097-003) at 4 °C with rocking for 15 min. Beads were then washed and resuspended with PBSB. Beads were profiled via flow cytometry using a BD Accuri C6 flow cytometer. Data analysis was performed with BD CSampler Plus software version 1.0.34.1 to obtain median fluorescence intensity (MFI) values, which are reported for each antibody across three or more replicate wells. Elotuzumab (purified using the low-throughput FPLC methodology described above), ixekizumab (FPLC purified as described above) and 4E10 (HIV Reagent Program, ARP-10091) are also included in each assay as controls.

### Lentivirus production

We produced SARS-CoV-2 Spike (D614G and Beta variants) pseudotyped lentiviral particles. Viral transfections were done in HEK293T cells (American Type Culture Collection, CRL-3216) using BioT (BioLand) transfection reagent. Six million cells were seeded in D10 media (DMEM + additives: 10% FBS, L-glutamate, penicillin, streptomycin and 10 mM HEPES) in 10-cm plates 1 day before transfection. A five-plasmid system was used for viral production, as described in Crawford et al.^64^. The Spike vector contained the 21-amino-acid truncated form of the SARS-CoV-2 Spike sequence from the Wuhan-Hu-1 strain of SARS-CoV-2 (GenBank: BCN86353.1) or the Beta variant of concern (GenBank: QUT64557.1). The other viral plasmids, used as previously described^64^, are pHAGE-Luc2-IRS-ZsGreen (NR-52516), HDM-Hgpm2 (NR-52517), pRC-CMV-Rev1b (NR-52519) and HDM-tat1b (NR-52518). These plasmids were added to D10 medium in the following ratios: 10 μg pHAGE-Luc2-IRS-ZsGreen, 3.4 μg FL Spike, 2.2 μg HDM-Hgpm2, 2.2 μg HDM-Tat1b and 2.2 μg pRC-CMV-Rev1b in a final volume of 1,000 μl.

Ebola GP-pseudotyped lentiviruses were produced using the same packaging (pHAGE-Luc2-IRS-ZsGreen) and helper plasmids (HDM-Hgpm2, HDM-Tat1b and pRC-CMV-Rev1b) but with the plasmid encoding full-length Ebola GP (GenBank: AAG40168.1).

After adding plasmids to medium, we added 30 μl of BioT to form transfection complexes. Transfection reactions were incubated for 10 min at room temperature, and then 9 ml of medium was added slowly. The resultant 10 ml was added to plated HEK cells from which the medium had been removed. Culture medium was removed 24 h after transfection and replaced with fresh D10 medium. Viral supernatants were harvested 72 h after transfection by spinning at 300*g* for 5 min, followed by filtering through a 0.45-μm filter. Viral stocks were aliquoted and stored at −80 °C until further use.

### Pseudovirus neutralization

The target cells used for infection in SARS-CoV-2 pseudovirus neutralization assays are from a HeLa cell line stably overexpressing human angiotensin-converting enzyme 2 (ACE2) as well as the protease known to process SARS-CoV-2: transmembrane serine protease 2 (TMPRSS2). Production of this cell line is described in detail by Rogers et al.^65^ with the addition of stable TMPRSS2 incorporation. ACE2/TMPRSS2/HeLa cells were plated 1 day before infection at 8,000 cells per well. For Ebola pseudovirus neutralization assays, HEK293T cells were seeded in 96-well plates 1 day before infection at 20,000 cells per well. Ninety-six-well, white-walled, white-bottom plates were used for neutralization assays (Thermo Fisher Scientific).

On the day of the assay, purified IgGs in 1× PBS were sterile filtered using a 0.22-μm filter. Dilutions of this filtered stock were made into sterile 1× Dulbecco’s PBS (DPBS) (Thermo Fisher Scientific), which was 5% by volume D10 medium. A virus mixture was made containing the virus of interest (for example, SARS-CoV-2) and D10 media (DMEM + additives: 10% FBS, L-glutamate, penicillin, streptomycin and 10 mM HEPES). Virus dilutions into media were selected such that a suitable signal would be obtained in the virus-only wells. A suitable signal was selected such that the virus-only wells would achieve a luminescence of at least >5,000,000 relative light units (RLU). Then, 60 μl of this virus mixture was added to each of the antibody dilutions to make a final volume of 120 μl in each well. Virus-only wells were made, which contained 60 μl of 1× DPBS and 60 μl of virus mixture. Cells-only wells were made, which contained 120 μl of D10 media.

The antibody/virus mixture was left to incubate for 1 h at 37 °C. After incubation, the medium was removed from the cells on the plates made 1 day prior. This was replaced with 100 μl of antibody/virus dilutions and incubated at 37 °C for approximately 24 h. Infectivity readout was performed by measuring luciferase levels. SARS-CoV-2 and Ebola pseudovirus neutralization assays were read out 48 h and 72 h after infection, respectively. Medium was removed from all wells, and cells were lysed by the addition of 100 μl of BriteLite assay readout solution (PerkinElmer) into each well. Luminescence values were measured using an Infinite 200 PRO Microplate Reader (Tecan) using i-control version 2.0 software (Tecan). Each plate was normalized by averaging the cells-only (0% infection) and virus-only (100% infection) wells. We used the neutcurve Python package version 0.5.7 to fit the normalized datapoints and to compute the IC_50_ values, which we report to two significant digits. To estimate measurement error, we computed the CV for each antibody–virus IC_50_ pair, and we report the mean CV for each virus in Supplementary Tables 5, 8 and 9.

### HLA binding prediction

As a proxy for predicting T-cell-mediated immunogenicity, we used NetMHCPan version 4.1 and NetMHCIIPan version 4.1 (ref. ^43^) to predict peptide binders to class I and class II HLA, respectively, across a number of alleles. For the class I analysis, we applied NetMHCPan with default parameters to the VH and VL sequences of the wild-type sequences as well as the VH and VL variant sequences listed in Fig. 3a. We considered all 9-mer peptides and predicted binding to HLA-A01:01, HLA-A02:01, HLA-A03:01, HLA-A24:02, HLA-A26:01, HLA-B07:02, HLA-B08:01, HLA-B27:05, HLA-B39:01, HLA-B40:01, HLA-B58:01 and HLA-B15:01. For each VH or VL sequence, we counted the number of peptides determined as ‘strong binders’ or ‘weak binders’ according to NetMHCPan. We then tested for a significant change in the number of binders between the evolved variant sequence and its corresponding wild-type using the binom_test function in scipy.stats. For the class II analysis, we similarly applied NetMHCIIPan with default parameters to the same set of VH and VL sequences. We considered all 15-mer peptides and predicted binding to DRB1_0101, DRB3_0101, DRB4_0101, DRB5_0101, HLA-DPA10103-DPB10101 and HLA-DQA10101-DQB10201. For each VH or VL sequence, we counted the number of peptides determined as ‘strong binders’ or ‘weak binders’ according to NetMHCIIPan. We then tested for a significant change in the number of binders between the evolved variant sequence and its corresponding wild-type using the binom_test function in scipy.stats.

### Computing frequency of changes to antibody protein sequences

We computed the frequency of residues involved in affinity-enhancing substitutions by aligning the wild-type VH and VL sequences of our antibodies to databases of protein sequences. The first database that we considered is UniRef90, where we used the same database release used to train ESM-1v. For each antibody protein sequence, we obtained the set of 10,000 sequences in UniRef90 that are closest to the antibody by sequence similarity based on Levenshtein distance (with the farthest sequences having between 18% and 47% sequence similarity). We computed sequence similarity using the FuzzyWuzzy Python package version 0.18.0. We then used MAFFT version 7.475 to perform multiple sequence alignment among the set of sequences. We used the alignment to compute amino acid frequencies at each site in the VH or VL sequence.

The second database that we considered is provided by the abYsis webtool, which also computes the frequency of amino acids at each position based on a multiple sequence alignment. We aligned VH and VL protein sequences using the default settings provided in the ‘Annotate’ tool, using the database of ‘All’ sequences as of 1 March 2022.

We also considered the frequency of affinity-enhancing substitutions conditioned on the corresponding V or J gene. We obtained all sequences and corresponding gene annotations from IMGT/LIGM-DB (the international ImMunoGeneTics information system, Laboratoire d’ImmunoGénétique Moléculaire database) (https://www.imgt.org/ligmdb/)^66^ as of 13 July 2022. For MEDI8852, MEDI8852 UCA, mAb114 and mAb114 UCA, we used the V and J gene annotations from the original publications^29,30^. For S309, REGN10987 and C143, we used the V and J gene annotations in CoV-AbDab (http://opig.stats.ox.ac.uk/webapps/covabdab/)^67–75^. For a given substitution, we obtained all corresponding V or J protein sequences, performed a multiple sequence alignment with MAFFT version 7.475 and used the resulting alignment to compute amino acid frequencies.

### Therapeutic antibody database evaluation and runtime benchmark

We downloaded 742 therapeutically relevant antibodies from the Thera-SAbDab database as of 26 February 2022 (http://opig.stats.ox.ac.uk/webapps/newsabdab/therasabdab/)^47^. For each antibody VH and VL sequence, we used the same procedure described above for computing consensus substitutions that have higher language model likelihood than wild-type. We measured the computational runtime using the time module in Python 3.8. Experiments were performed with an Advanced Micro Devices EPYC Rome 7502P 2.5-GHz CPU and an Nvidia Ampere A40 48GB GPU.

### Natural protein evaluation and benchmarking based on scanning mutagenesis data

We evaluated the ability for the language models and algorithms used in our study to guide efficient evolution in other settings beyond antibodies. We used deep mutational scanning (DMS) datasets to validate that our approach would enable a researcher to acquire high-fitness variants. We used all DMS datasets from the benchmarking study by Livesey and Marsh^48^ with 90% or higher coverage of all single-residue substitutions; variants that were not measured were excluded from the analysis. We also used a scanning mutagenesis dataset generated by Markin et al.^8^ that measured Michaelis–Menten kinetics of all single-site glycine or valine substitutions to the bacterial enzyme PafA; for this dataset, any language-model-recommended substitutions that did not involve glycine or valine substitutions were excluded from the analysis. We applied a cutoff for each dataset to binarize sequences as high-fitness or low-fitness variants (cutoffs are provided in Supplementary Table 13); we then compared enrichment of high-fitness variants among the language-model-recommended variants to the background frequency of high-fitness variants among all single-residue substitutions. For these proteins, as with our antibody experiments, we chose values of *k* that result in a small number (~10^1^) of acquired substitutions: we used *α* = 1 and *k* = 2 for all proteins except those where this resulted in $|{{{\mathcal{A}}}}|$ ≤5, in which case we set *k* = 1 (and additionally *α* = 0.5 for infA).

To quantify the statistical significance of an enrichment, we assumed that the null distribution of the number of high-fitness, language-model-recommended variants was given by a hypergeometric distribution parameterized by the number of language-model-recommended variants $|{{{\mathcal{A}}}}|$, the number of high-fitness variants among the all single-residue substitutions and the total number of single-residue substitutions considered, which we used to compute a one-sided *P* value. We used the hypergeometric calculator at https://stattrek.com/online-calculator/hypergeometric.aspx.

To test the relationship between likelihood stringency and the fraction of high-fitness substitutions, we also performed a small-scale parameter sweep varying the cutoff values *α* and *k* and computing (1) the percentage fraction of high-fitness substitutions in ${{{\mathcal{A}}}}$; (2) the maximum fitness value of a variant in ${{{\mathcal{A}}}}$ divided by the maximum fitness value of a variant across the full mutational scan; and (3) the maximum fitness value of a variant in ${{{\mathcal{A}}}}$ divided by the 99th percentile of the fitness values across the full mutational scan; before this normalization, the raw fitness values are also linearly scaled to take values between 0 and 1, inclusive. Normalized values, the number of acquired variants $|{{{\mathcal{A}}}}|$ and the parameter combinations are plotted in Extended Data Fig. 4.

We also tested how well alternative methods for ranking substitutions would be able to suggest high-fitness variants. To enable a direct comparison to the language model consensus strategy described above, we selected the same number of substitutions and kept all other parameters fixed while only varying the method used to rank substitutions. We used the benchmarking results obtained by Livesey and Marsh^48^ enabling us to test 46 different methods for ranking substitutions, which use evolutionary information, biophysical properties of amino acids or protein structure information; these methods are described in greater detail in Table EV1 of ref. ^48^. We also tested how well using the summed log-likelihood ratios across all ESM language models (that is, computing $\mathop {\sum}\nolimits_j {\left( {\log p_j\left( {x_i^\prime |x} \right) - \log p_j\left( {x_i|{{{\mathbf{x}}}}} \right)} \right)}$ at each site *i* and substitution $x_i^\prime$) would compare to the consensus strategy. For each DMS dataset, we computed the number of high-fitness mutations that were acquired by each of these 47 benchmark methods (Extended Data Fig. 5); we broke any ties in variant effect predictor scores by randomly selecting substitutions and computing the average number of high-fitness variants over 100 random seeds. We aggregated results across DMS datasets by ranking methods within each DMS (averaging the ranks that would have been assigned to tied values) and computed the mean rank across the eight DMS datasets (Extended Data Fig. 5 and Supplementary Data 5).

### Reporting Summary

Further information on research design is available in the Nature Portfolio Reporting Summary linked to this article.

## Online content

Any methods, additional references, Nature Portfolio reporting summaries, source data, extended data, supplementary information, acknowledgements, peer review information; details of author contributions and competing interests; and statements of data and code availability are available at 10.1038/s41587-023-01763-2.

### Supplementary information

Supplementary Information Supplementary Tables 1–13 and Supplementary Information.

Reporting Summary

Supplementary Data 1 Experimental *K*_d_, IC_50_ and *T*_m_ values across seven antibody-directed evolution campaigns.

Supplementary Data 2 MFI values for polyspecificity experiments and predicted MHC-binding peptides for immunogenicity experiments.

Supplementary Data 3 Benchmark results for comparison to other sequence-based methods.

Supplementary Data 4 Language-model-recommended amino acid substitutions for 742 therapeutic antibodies.

Supplementary Data 5 Mean rank values of 48 methods across DMS benchmarking experiments.

Supplementary Data 6 List of oligonucleotide sequences used in this paper.

Supplementary Code Relevant code and scripts described in this paper.

## Data Availability

Raw data for this study have been deposited to Zenodo at 10.5281/zenodo.6968342. *K*_d_, IC_50_ and *T*_m_ values across replicate experiments are available as Supplementary Data 1. Median fluorescence intensity values for the polyspecificity experiments are available as Supplementary Data 2. Experimental values for our benchmarking of sequence-based methods and results from our UniRef90 parameter sweeps are available as Supplementary Data 3. High-likelihood amino acid substitutions for 742 therapeutic antibodies are available as Supplementary Data 4. Mean rank values for our deep mutational scanning benchmark experiments are available as Supplementary Data 5. A list of oligonucleotides used in the study is provided as Supplementary Data 6. We also make use of the following publicly available databases and datasets: • UniProt: https://www.uniprot.org/ • UniRef50 2018_03 (ref. ^23^): https://ftp.uniprot.org/pub/databases/uniprot/previous_releases/release-2018_03/uniref/ • UniRef90 2020_03 (ref. ^23^): https://ftp.uniprot.org/pub/databases/uniprot/previous_releases/release-2020_03/uniref/ • abYsis^44^: http://www.abysis.org/abysis/ • IMGT/LIGM-DB^66^: https://www.imgt.org/IMGTindex/LIGM-DB.php • Thera-SAbDab^47^: https://opig.stats.ox.ac.uk/webapps/newsabdab/therasabdab/search/ • Livesey and Marsh benchmarking dataset^48,68–75^.

## Code availability

We provide open-source code that enables a user to easily and quickly evaluate the language models on a sequence of interest. We implement this as a simple call to a Python script with the wild-type sequence as the main argument, which is available at https://github.com/brianhie/efficient-evolution. Code and scripts used in this study are available as Supplementary Code and have been deposited to Zenodo at 10.5281/zenodo.6977562.
