# Supplementary material for: Efficient evolution of human antibodies from general protein language models
Source: Nat Biotechnol. 2023 Apr 24;42(2):275–83. doi: 10.1038/s41587-023-01763-2 (PMC10869273; doi:10.1038/s41587-023-01763-2)
Supplement: Supplementary file 1 — Supplementary Tables 1–13 and Supplementary Information. [file 41587_2023_1763_MOESM1_ESM.pdf]

nature biotechnology

Article https://doi.org/10.1038/s41587-023-01763-2

Efficient evolution of human antibodies from general protein language models

In the format provided by the authors and unedited

Supplementary Tables 1-13

Antibody	Matured?	Source	Virus	Screened antigen	Round-1 variants with improved binding	Round-1 variants with preserved binding	Best affinity improvement (antigen)	Best neutralization improvement (virus)
MEDI8852 [29]	Yes (in vivo + in vitro)	Patient-derived + in vitro evolution	Influenza A	HA H4 Hubei	2 / 14 = 14%	13 / 14 = 93%	7-fold (HA H7 HK17)	ND
MEDI8852 UCA [29]	No	Germline-inferred	Influenza A	HA H1 Solomon	3 / 8 = 38%	8 / 8 = 100%	23-fold (HA H4 Hubei)	ND
mAb114 [30]	Yes	Patient-derived	Ebolavirus	GP	7 / 13 = 54%	13 / 13 = 100%	3.4-fold (GP)	1.5-fold (GP pseudotyped lentivirus)
mAb114 UCA [30]	No	Germline-inferred	Ebolavirus	GP	6 / 9 = 67%	7 / 9 = 78%	160-fold (GP)	>100-fold (GP pseudotyped lentivirus)
S309 [31]	Yes	Patient-derived	Sarbecovirus	Wuhan-Hu-1 S-6P	2 / 10 = 20%	9 / 10 = 90%	1.7-fold (Beta S-6P)	ND
REGN10987 [32]	Yes	Patient-derived	SARS-CoV-2	Beta S-6P	2 / 8 = 25%	8 / 8 = 100%	1.3-fold (Beta S-6P)	2-fold (Beta pseudotyped lentivirus)
C143 [39]	No	Patient-derived	SARS-CoV-2	Beta S-6P	10 / 14 = 71%	10 / 14 = 71%	13-fold (Beta S-6P)	31-fold (Beta pseudotyped lentivirus)

Supplementary Table 1: Summary of antibodies considered in this study.

Information on the antibodies considered in each of our directed evolution campaigns. Matured indicates extensive somatic hypermutation from germline (and, in the case of MEDI8852, additional in-vitro affinity maturation). Source indicates how the antibody sequence was obtained; germline-inferred sequences were obtained from the original publications. Improved binding is

defined as a 1.1-fold improvement or higher from wildtype. Preserved binding is defined as a sub-micromolar K_d for the screened antigen. ND: not determined.

Round	Design	Region	H4 Hubei Fab K_d (nM)	H7 HK16 Fab K_d (nM)	H7 HK17 Fab K_d (nM)
	MEDI8825 WT		0.60	6.2	0.21
Round 1	VH D27F	HFR1	1.1	7.8	ND
	VH V35Y	CDR-H1	0.83	9.2	ND
	VH S44G	HFR2	0.66	6.0	ND
	VH T53I	CDR-H2	0.72	11	ND
	VH W59S	CDR-H2	0.91	7.6	ND
	VH E65P	CDR-H2	**0.40**	**2.8**	**0.10**
	VH N74S	HFR3	0.61	7.2	ND
	VH M117Y	CDR-H3	**0.43**	4.5	ND
	VL T25A	CDR-L1	1.7	19	ND
	VL L29V	CDR-L1	5.2	71	ND
	VL T33L	CDR-L1	3.6	35	ND
	VL G55A	CDR-L2	0.60	5.7	ND
	VL R92D	CDR-L3	NB	25	ND
	VL G95P	LFR4	0.94	5.1	ND
Round 2	VH E65P-M117Y	Multi	**0.31**	**2.5**	**0.030**
		Error (mean CV):	± 12%	± 14%	± 34%

Supplementary Table 2: MEDI8852 variants.

Variants tested across two rounds of directed evolution, the corresponding Kabat-annotated regions, and K_d values for three HA antigens. The wildtype row is highlighted in gray; variants with improved affinity against the screened antigen (fold improvement > 1.1) are highlighted in blue; values with one-sided *t*-test $P < 0.05$ compared to wildtype are bolded. NB: no binding; ND: not determined; CV: coefficient of variation.

Antigen (Group)	Antibody	Fab K_d (nM)
H1 Caledonia (Group 1)	MEDI8852	<0.001
	VH E65P	<0.001
	VH E65P-M117Y	<0.001
H1 Solomon (Group 1)	MEDI8852	<0.001
	VH E65P	<0.001
	VH E65P-M117Y	<0.001
H2 Japan (Group 1)	MEDI8852	<0.001
	VH E65P	<0.001
	VH E65P-M117Y	<0.001
H3 Panama (Group 2)	MEDI8852	<0.001
	VH E65P	<0.001
	VH E65P-M117Y	<0.001
H3 Victoria (Group 2)	MEDI8852	<0.001
	VH E65P	<0.001
	VH E65P-M117Y	<0.001
H4 Hubei (Group 2)	MEDI8852	0.70
	VH E65P	0.40
	VH E65P-M117Y	0.31
H5 Vietnam (Group 1)	MEDI8852	<0.001
	VH E65P	<0.001
	VH E65P-M117Y	<0.001
H7 HK16 (Group 2)	MEDI8852	6.2
	VH E65P	2.8
	VH E65P-M117Y	2.5
H7 HK17 (Group 2)	MEDI8852	0.21
	VH E65P	0.10
	VH E65P-M117Y	0.030

Supplementary Table 3: Binding to Group 1 and Group 2 HAs.

Binding affinity between MEDI8852 WT Fab and three variant Fabs against a panel of nine HAs. A K_d of <0.001 indicates an interaction with no observed dissociation when measured via BLI.

Round	Design	Region	H1 Solomon IgG K_d app. (nM)	H1 Solomon Fab K_d (nM)	H4 Hubei IgG K_d app. (nM)	H4 Hubei Fab K_d (μM)	H7 HK17 K_d (nM)
	MEDI8852 UCA WT		2.0	140	740	29	IgG app. K_d: 17<br>Fab K_d: 4400
Round 1	VH S44G	HFR2	2.1	ND	ND	ND	ND
	VH T53I	CDR-H2	2.1	ND	ND	ND	ND
	VH K58S	CDR-H2	**1.5**	**110**	ND	**2.5**	ND
	VH V65P	CDR-H2	**1.7**	ND	ND	ND	ND
	VH N74S	HFR3	2.6	ND	ND	ND	ND
	VH P75R	HFR3	2.0	ND	ND	ND	ND
	VL N34A	CDR-L1	8.2	ND	ND	ND	ND
	VL G95P	LFR4	**1.3**	**65**	ND	**17**	ND
Round 2	VH K58S-V65P	CDR-H2	**1.3**	ND	**80**	ND	ND
	VH K58S-P75R	Multi	**1.2**	ND	**47**	ND	ND
	VH V65P-P75R	Multi	**1.5**	ND	**570**	ND	ND
	VH K58S-V65P-P75R	Multi	**1.2**	ND	**72**	ND	ND
	VH K58S / VL G95P	Multi	**0.96**	**56**	**36**	**3.1**	ND
	VH V65P / VL G95P	Multi	**0.93**	ND	**100**	ND	ND
	VH P75R / VL G95P	Multi	**0.95**	ND	**110**	ND	ND
	VH K58S-V65P / VL G95P	Multi	**0.78**	**50**	**30**	**1.3**	IgG app. K_d: **5.6**<br>Fab K_d: **810**
	VH K58S-P75R / VL G95P	Multi	**0.88**	ND	**45**	ND	ND
	VH V65P-P75R / VL G95P	Multi	**0.87**	ND	**110**	ND	ND
	VH K58S-V65P-P75R / VL G95P	Multi	**0.79**	**55**	**42**	**1.5**	ND
		Error (mean CV):	± 11%	± 8.6%	± 10%	± 16%	± 13%

Supplementary Table 4: MEDI8852 UCA variants.

Variants tested across two rounds of directed evolution, the corresponding Kabat-annotated regions, and K_d values for three HA antigens. In Round 2, all possible combinations involving K58S, V65P, P75R in the VH and G95P in the VL were made. The wildtype row is highlighted

in gray; variants with improved affinity against the screened antigen (fold improvement > 1.1) are highlighted in blue; values with one-sided *t*-test $P < 0.05$ compared to wildtype are bolded . K_d app.: K_d apparent; ND: not determined; CV: coefficient of variation.

Round	Design	Region	GP Fab K_d (nM)	Ebola GP pseudotyped lentivirus IC_{50} (nM)
	mAb114 WT		0.21	1.4
Round 1	VH M31S	CDR-H1	0.13	ND
	VH I41P	HFR2	0.33	ND
	VH D42G	HFR2	0.18	ND
	VH A68T	HFR3	0.10	ND
	VH E72D	HFR3	0.13	ND
	VH S79Y	HFR3	0.12	ND
	VH I113T	HFR4	**0.071**	ND
	VL I19V	LFR1	0.23	ND
	VL F29I	CDR-L1	0.46	ND
	VL V43A	LFR2	**0.068**	ND
	VL S49Y	LFR2	0.58	ND
	VL H70D	LFR3	0.27	ND
	VL N90Q	CDR-L3	4.0	ND
Round 2	VH D42G-A68T-S79Y	Multi	0.19	1.2
	VH I41P-D42G-A68T-S79Y-I113T	Multi	0.19	1.2
	VH A68T-I113T / VL V43A	Multi	0.17	1.1
	VH A68T-E72D-S79Y-I113T / VL V43A	Multi	**0.076**	1.2
	VH D42G-A68T-S79Y / VL V43A	Multi	**0.061**	**0.96**
	VH I41P-D42G-A68T-S79Y-I113T / VL V43A	Multi	**0.069**	**1.0**
		Error (mean CV):	± 41%	± 18%

Supplementary Table 5: mAb114 variants.

Variants tested across two rounds of directed evolution, the corresponding Kabat-annotated regions, and K_d values for ebolavirus GP. Neutralization IC_{50} values were also determined for mAb114 WT and all Round-2 variant IgGs against GP-pseudotyped lentivirus. The wildtype row is highlighted in gray; variants with improved affinity against the screened antigen (fold

improvement > 1.1) are highlighted in blue; values with one-sided *t*-test $P < 0.05$ compared to wildtype are bolded. ND: not determined; CV: coefficient of variation.

Round	Design	Region	GP IgG K_d app. (μM)	GP Fab K_d (μM)
	mAb114 UCA WT		7.9	75
Round 1	VH T41P	HFR2	2.9	ND
	VH A54G	CDR-H2	NB	ND
	VH P60A	CDR-H2	NB*	ND
	VH G61D	CDR-H2	2.8	ND
	VH E72D	HFR3	3.6	ND
	VH G88E	HFR3	**3.0**	ND
	VH V96A	HFR3	2.2	ND
	VL V43A	LFR2	**1.2**	ND
	VL K90Q	CDR-L3	13	ND
Round 2	VH P60A-G61D	CDR-H2	**2.4**	ND
	VH T41P-P60A-G61D-E72D-G88E-V96A	Multi	**0.63**	ND
	VH G88E / VL V43A	Multi	**0.26**	ND
	VH V96A / VL V43A	Multi	**0.46**	ND
	VH P60A-G61D / VL V43A	Multi	**0.097**	**0.48**
	VH P60A-G61D-G88E-V96A / VL V43A	Multi	**0.27**	ND
	VH T41P-P60A-G61D-E72D-G88E-V96A /VL V43A	Multi	**0.32**	ND
Error (mean CV):			± 42%	± 3.0%

Supplementary Table 6: mAb114 UCA variants.

Variants tested across two rounds of directed evolution, the corresponding Kabat-annotated regions, and K_d values (for both IgG and Fab versions) for ebolavirus GP. The wildtype row is highlighted in gray; variants with improved affinity against the screened antigen (fold improvement > 1.1) are highlighted in blue; values with one-sided *t*-test $P < 0.05$ compared to wildtype are bolded. An asterisk (*) indicates examples where some binding was observed but

BLI data were not suitable for fitting. K_d app.: K_d apparent; ND: not determined; CV: coefficient of variation.

Round	Design	Region	W1 S-6P Fab K_d (nM)	Beta S-6P Fab K_d (nM)	Omicron RBD Fab K_d (nM)
	S309 WT		2.5	2.4	14
	Sotrovimab (VH N55Q)	CDR-H2	2.4	2.0	17
Round 1	VH P28T	HFR1	4.3	ND	ND
	VH T77N	HFR3	2.8	ND	ND
	VH G79A	HFR3	2.8	ND	ND
	VH R84S	HFR3	2.7	ND	ND
	VH R85S	HFR3	3.1	ND	ND
	VH R87T	HFR3	2.1	ND	ND
	VL T28S	CDR-L1	**2.1**	ND	ND
	VL T32N	CDR-L1	2.7	ND	ND
	VL S95V	CDR-L3	NE	ND	ND
	VL L96P	CDR-L3	2.7	ND	ND
Round 2	VH T77N-G79A-R84S	HFR3	3.2	ND	ND
	VH T77N-G79A-R84S-R85S	HFR3	3.1	ND	ND
	VL T28S-T32N	CDR-L1	3.0	ND	ND
	VH G79A / VL T28S	Multi	**2.0**	**1.4**	15
	VH R84S / VL T28S	Multi	2.5	ND	ND
	VH R87T / VL T28S	Multi	2.5	ND	ND
	VH T77N-G79A-R84S / VL T28S	Multi	3.3	ND	ND
	VH T77N-G79A-R84S / VL T28S-T32N	Multi	3.5	ND	ND
	VH T77N-G79A-R84S-R85S / VL T28S-T32N	Multi	4.3	ND	ND
		Error (mean CV):	± 19%	± 14%	± 16%

Supplementary Table 7: S309 variants.

Variants tested across two rounds of directed evolution, the corresponding Kabat-annotated regions, and K_d values for antigens from three SARS-CoV-2 variants. The wildtype row is highlighted in gray; variants with improved affinity against the screened antigen (fold

improvement > 1.1) are highlighted in blue; values with one-sided *t*-test $P < 0.05$ compared to wildtype are bolded . W1: Wuhan-Hu-1; NE: no expression; ND: not determined; CV: coefficient of variation.

Round	Design	Region	Beta S-6P IgG K_d app. (nM)	Beta S-6P Fab K_d (nM)	Omicron RBD IgG K_d app. (μM)	D614G-pseudotyped lentivirus IC_{50} (ng/mL)	Beta-pseudotyped lentivirus IC_{50} (ng/mL)
	REGN10987 WT		0.091	14	0.70 (Fab K_d: 23)	1.1	1.4
Round 1	VH R16G	HFR1	0.073	**11**	0.51	0.83	**0.73**
	VH S98R	HFR3	0.41	ND	ND	ND	ND
	VH V108D	CDR-H3	0.076	26	ND	ND	ND
	VL S82A	LFR3	0.063	17	ND	ND	ND
	VL N91C	CDR-L3	0.053	12	ND	ND	ND
	VL N91S	CDR-L3	0.066	27	ND	ND	ND
	VL L93Y	CDR-L3	0.30	ND	ND	ND	ND
	VL I96S	CDR-L3	0.072	15	ND	ND	ND
Round 2	VH R16G-V108D	Multi	ND	25	2.2	ND	ND
	VL S82A-N91C-I96S	Multi	ND	**12**	0.48	1.0	**0.87**
	VH R16G / VL S82A	Multi	ND	**12**	0.88	1.0	**0.78**
	VH R16G / VL N91C	Multi	ND	23	0.42 (Fab K_d: **4.5**)	ND	ND
	VH R16G / VL I96S	Multi	ND	13	1.2	1.2	**0.79**
	VH R16G-V108D / VL S82A-N91C-I96S	Multi	ND	36	2.5	ND	ND
		Error (mean CV):	NA	± 32%	± 19%	± 30%	± 15%

Supplementary Table 8: REGN10987 variants.

Variants tested across two rounds of directed evolution, the corresponding Kabat-annotated regions, and K_d values for antigens from two SARS-CoV-2 variants. Round-1 variants were pre-screened as IgGs with a single replicate before testing the highest-avidity variants as Fabs and with multiple replicates. Neutralization IC_{50} values were also determined for REGN10987 WT and selected affinity-enhancing variant IgGs against D614G- and Beta-pseudotyped lentivirus. The wildtype row is highlighted in gray; variants with improved affinity against the screened antigen (fold improvement > 1.1) are highlighted in blue; values with one-sided *t*-test $P < 0.05$

compared to wildtype are bolded. K_d app.: K_d apparent; NA: not applicable; ND: not determined; CV: coefficient of variation.

Round	Design	Region	Beta S-6P IgG K_d app. (nM)	W1 S-6P IgG K_d app. (nM)	Omicron IgG K_d app. (µM)	D614G pseudotyped lentivirus IC_{50} (ng/mL)	Beta pseudotyped lentivirus IC_{50} (ng/mL)
	C143 WT		450	39	610	1400	5800
Round 1	VH V29F	HFR1	**140**	ND	ND	ND	ND
	VH K32Y	CDR-H1	32000	ND	ND	ND	ND
	VH L51Y	CDR-H2	180	ND	ND	ND	ND
	VH D57T	CDR-H2	NB*	ND	ND	ND	ND
	VH A77T	HFR3	170	ND	ND	ND	ND
	VH G91A	HFR3	170	ND	ND	ND	ND
	VL N27S	CDR-L1	130	ND	ND	ND	ND
	VL T33N	CDR-L1	**37**	ND	ND	**200**	**870**
	VL L34Y	CDR-L1	200	ND	ND	ND	ND
	VL Y41H	LFR2	**110**	ND	ND	ND	ND
	VL G53V	CDR-L2	**29**	ND	ND	**110**	**180**
	VL S57P	CDR-L2	NB*	ND	ND	ND	ND
	VL G82A	LFR3	NB*	ND	ND	ND	ND
	VL A96S	CDR-L3	250	ND	ND	ND	ND
Round 2	VH L51Y-A77T-G91A	Multi	NB*	ND	ND	ND	ND
	VL T33N-G53V	Multi	**34**	30	**160**	**74**	**290**
	VL N27S-T33N-L34Y-G53V	Multi	520	ND	ND	ND	ND
	VL N27S-T33N-L34Y-Y41H-G53V-S57P-G82A	Multi	800	ND	ND	ND	ND
	VH L51Y-A77T-G91A / VL T33N	Multi	NB*	ND	ND	ND	ND
	VH L51Y-A77T-G91A / VL N27S-T33N-L34Y-G53V	Multi	320	ND	ND	ND	ND
		Error (mean CV):	± 48%	± 26%	± 24%	± 21%	± 36%

Supplementary Table 9: C143 variants.

Variants tested across two rounds of directed evolution, the corresponding Kabat-annotated regions, and K_d values for antigens from three SARS-CoV-2 variants. Neutralization IC_{50} values were also determined for C143 WT and selected affinity-enhancing variant IgGs against D614G-

and Beta-pseudotyped lentivirus. The wildtype row is highlighted in gray; variants with improved affinity against the screened antigen (fold improvement > 1.1) are highlighted in blue; values with one-sided *t*-test $P < 0.05$ compared to wildtype are bolded. An asterisk (*) indicates examples where some binding was observed but BLI data were not suitable for fitting. W1: Wuhan-Hu-1; K_d app.: K_d apparent; NB: no binding; ND: not determined; CV: coefficient of variation.

Antibody	Affinity-enhancing substitution	UniRef90 (training dataset)					abYsis			
		Wildtype residue frequency	Mutant residue frequency	Top residue	Top residue frequency	Notes	Wildtype residue frequency	Mutant residue frequency	Top residue	Top residue frequency
MEDI8852	VH E65P	6%	32%	D	35%		14%	22%	D	32%
	VH M117Y	<1%	59%	-	-		<1%	49%	-	-
MEDI8852 UCA	VH K58S	8%	3%	T	67%	Uncommon to rare, site-independent rank 8	2%	30%	G	35%
	VH V65P	1%	32%	D	36%		2%	22%	D	32%
	VL G95P	44%	2%	G	44%	Common to rare, site-independent rank 2728	99%	<1%	G	99%
mAb114	VH M31S	<1%	45%	-	-		<1%	48%	-	-
	VH D42G	1%	88%	-	-		<1%	91%	-	-
	VH A68T	1%	88%	-	-		1%	85%	-	-
	VH E72D	4%	91%	-	-		2%	93%	-	-
	VH S79Y	8%	74%	-	-		16%	67%	-	-
	VH I113T	2%	69%	-	-		2%	89%	-	-
	VL V43A	4%	50%	-	-		2%	57%	-	-
mAb114 UCA	VH T41P	1%	94%	-	-		1%	92%	-	-
	VH G61D	2%	57%	-	-		1%	32%	-	-
	VH E72D	4%	92%	-	-		2%	93%	-	-
	VH G88E	1%	85%	-	-		2%	67%	-	-
	VH V96A	6%	77%	-	-		5%	85%	-	-
	VL V43A	4%	50%	-	-		2%	57%	-	-
S309	VH R87T	37%	30%	R	37%		39%	41%	-	-
	VL T28S	1%	54%	-	-		2%	77%	-	-
REGN10987	VH R16G	5%	68%	-	-		6%	31%	-	-
	VL N91C	2%	2%	Q	39%	Rare to rare, site-independent rank 85	1%	4%	Q	52%
C143	VH V29F	3%	78%	-	-		4%	69%	-	-
	VH L51Y	4%	18%	-	-		1%	<1%	I	85%

	VH A77T	1%	56%	-	-		<1%	62%	-	-
	VH G91A	4%	95%	-	-		2%	97%	-	-
	VL N27S	11%	53%	-	-		3%	77%	-	-
	VL T33N	2%	44%	-	-		12%	36%	-	-
	VL L34Y	4%	34%	-	-		2%	59%	-	-
	VL Y41H	<1%	6%	K	59%	Rare to uncommon, site-independent rank 66	<1%	5%	K	67%
	VL G53V	2%	10%	A	30%	Rare to uncommon, site-independent rank 247	4%	12%	A	40%
	VL A96S	2%	57%	-	-		2%	36%	-	-

Supplementary Table 10: Originality of affinity-enhancing substitutions.

Each row corresponds to an amino acid substitution that enhances the binding affinity of its corresponding variant antibody, and some of which also enhance affinity in combination with other substitutions. We computed frequencies of amino acid substitutions among natural sequences using two datasets, UniRef90 and abYsis (**Methods**); UniRef90 was the sequence database used to train the language models in our algorithm and abYsis is a separate, curated database of natural antibody sequences. The "wildtype residue frequency" indicates the percentage of sequences in a multiple sequence alignment with the same residue as wildtype at the given position; the "mutant residue frequency" is the same statistic except for the mutant residue. The "top residue" indicates the amino acid with the highest frequency observed at the given site, the "top residue frequency" indicates the percentage of sequences that contain the top residue at the given site, and dashes indicate settings in which the mutant residue is also the top residue. Substitutions with frequencies up to 5% are considered "rare," those with frequencies above 5% and up to 10% are considered "uncommon," and those above 10% are considered

“common.” Blue shading indicates substitutions to rare or uncommon residues according to frequency information from either UniRef90 or abYsis. The “site-independent rank” indicates the rank of the corresponding rare or uncommon substitution when ordering all possible single-residue substitutions according to the likelihood ratio to wildtype under a UniRef90-based site-independent model (**Supplementary Table 12**), where a rank of 1 indicates the highest likelihood ratio.

Antibody	Affinity-enhancing substitution	IMGT germline statistics						
		Gene	Number of database sequences	Wildtype residue frequency	Mutant residue frequency	Top residue	Top residue frequency	Notes
MEDI8852	VH E65P	IGHV6-1	297	4%	3%	V	77%	Rare to rare
	VH M117Y	IGHJ3	4546	2%	51%	-	-	
MEDI8852 UCA	VH K58S	IGHV6-1	297	83%	<1%	K	83%	Common to rare
	VH V65P	IGHV6-1	297	74%	3%	V	74%	Common to rare
	VL G95P	IGKJ1	2019	94%	<1%	G	94%	Common to rare
mAb114	VH M31S	IGHV3-13	75	1%	60%	-	-	
	VH D42G	IGHV3-13	75	1%	86%	-	-	
	VH A68T	IGHV3-13	75	1%	86%	-	-	
	VH E72D	IGHV3-13	75	69%	22%	E	69%	
	VH S79Y	IGHV3-13	75	5%	83%	-	-	
	VH I113T	IGHJ4	8345	2%	82%	-	-	
	VL V43A	IGKV1-27	83	86%	7%	V	86%	Common to uncommon
mAb114 UCA	VH T41P	IGHV3-13	75	60%	17%	T	60%	
	VH G61D	IGHV3-13	75	55%	31%	G	55%	
	VH E72D	IGHV3-13	75	69%	22%	E	69%	
	VH G88E	IGHV3-13	75	66%	22%	G	66%	
	VH V96A	IGHV3-13	75	16%	63%	-	-	
	VL V43A	IGKV1-27	83	86%	7%	V	86%	Common to uncommon
S309	VH R87T	IGHV1-18	597	62%	16%	R	62%	
	VL T28S	IGKV3-20	909	3%	86%	-	-	
REGN10987	VH R16G	IGHV3-30	330	64%	19%	R	64%	
	VL N91C	IGLV2-14	301	4%	2%	S	81%	Rare to rare
C143	VH V29F	IGHV3-66	44	75%	5%	V	75%	Common to rare
	VH L51Y	IGHV3-66	44	7%	<1%	I	84%	Uncommon to rare
	VH A77T	IGHV3-66	44	2%	87%	-	-	
	VH G91A	IGHV3-66	44	5%	82%	-	-	

	VL N27S	IGLV2-23	116	3%	91%	-	-	
	VL T33N	IGLV2-23	116	<1%	91%	-	-	
	VL L34Y	IGLV2-23	116	75%	6%	L	75%	Common to uncommon
	VL Y41H	IGLV2-23	116	7%	91%	-	-	
	VL G53V	IGLV2-23	116	37%	57%	-	-	
	VL A96S	IGLV2-23	116	2%	77%	-	-	

Supplementary Table 11: Germline frequencies of affinity-enhancing substitutions. Each row corresponds to an amino acid substitution that enhances the binding affinity of its corresponding variant antibody, and some of which also enhance affinity in combination with other substitutions. We computed frequencies of amino acid substitutions based on IMGT germline sequences, where for each substitution, we restrict the multiple sequence alignment to only include sequences from its corresponding V or J gene, which are listed in the "gene" column (**Methods**). The "number of database sequences" indicates the number of germline sequences found for that gene in IMGT (the denominator of frequency values). The "wildtype residue frequency" indicates the percentage of sequences in a multiple sequence alignment with the same residue as wildtype at the given position; the "mutant residue frequency" is the same statistic except for the mutant residue. The "top residue" indicates the amino acid with the highest frequency observed at the given site, the "top residue frequency" indicates the percentage of sequences that contain the top residue at the given site, and dashes indicate settings in which the mutant residue is also the top residue. Substitutions with frequencies up to 5% are considered "rare," those with frequencies above 5% and up to 10% are considered "uncommon," and those above 10% are considered "common." Blue shading indicates substitutions to rare or uncommon residues according to frequency information from IMGT germline sequences.

Antibody	Metric	Method				
		This study	abYsis	UniRef90	AbLang	Sapiens
MEDI UCA	Fraction with improved binding	3/8 = 38%	0/8 = 0%	2/8 = 25%	0/8 = 0%	1/8 = 13%
	Median fold improvement	1.2	1.0	1.1	0.45	0.95
	Maximum fold improvement	1.7	1.2	1.5	1.1	1.5
mAb114 UCA	Fraction with improved binding	6/9 = 67%	5/9 = 56%	5/9 = 56%	1/9 = 11%	1/9 = 11%
	Median fold improvement	2.2	1.9	1.9	0.78	0.98
	Maximum fold improvement	6.0	6.0	4.8	3.1	3.1
C143	Fraction with improved binding	3/14 = 21%	0/14 = 0%	0/14 = 0%	1/14 = 7%	1/14 = 7%
	Median fold improvement	1.3	0.86	0.22	0.79	0.81
	Maximum fold improvement	12	1.7	1.3	8.1	8.1

Supplementary Table 12: Benchmarking results comparing sequence-based methods for proposing substitutions.

We compared the changes in avidity of substitutions recommended by four sequence-based baseline methods (abYsis, UniRef90, AbLang, and Sapiens) to those of the substitutions used in the first round of our evolutionary campaigns (this study). Summary statistics are: (1) the fraction with improved binding, defined as a change in IgG apparent K_d with nominal one-sided t-test $P < 0.05$; (2) the median fold improvement across all substitutions; and (3) the maximum fold improvement across all substitutions. The best values in each category are shaded light blue. The full set of benchmark results is provided in **Supplementary Data 3**.

**Protein**	**Reference**	**Organism**	**Fitness setting**	**Cutoff**	**Sample successes**	**Sample size**	**Population successes**	**Population size**	**Hit rate (%)**	**Background (%)**	**Hypergeo. *P***
ADRB2	Jones et al., 2019 [72]	Human	Signal transduction + pathway reporter	> 2.8	2	9	914	7800	22%	12%	0.28
β-lactamase	Stiffler et al, 2015 [73]	Bacteria	Antibiotic resistance (ampicillin, 2500 ug/mL)	> 0.01	4	10	393	4978	40%	7.9%	0.0055
Env	Haddox et al., 2016 [74]	Virus	Viral replication fitness	> 0.1	7	31	748	12863	23%	5.8%	0.0017
HA H1	Doud and Bloom, 2016 [75]	Virus	Viral replication fitness	> 0.1	5	32	645	10716	16%	6.0%	0.041
HA H3	Lee et al., 2018 [76]	Virus	Viral replication fitness	> 0.1	5	16	714	10754	31%	6.6%	0.0030
infA	Kelsic et al., 2016 [77]	Bacteria	Competitive growth	> 0.98	5	10	305	1368	50%	22%	0.050
MAPK1	Brenan et al., 2016 [78]	Human	Competitive growth (SCH772984)	> 2.5	1	13	77	6810	7.7%	1.1%	0.14
P53	Giacomelli et al, 2018 [79]	Human	Competitive growth (etoposide)	> 1	2	17	905	7448	12%	12%	0.63
PafA	Markin et al., 2021 [8]	Bacteria	K_{cat}/K_M	$P < 0.01$, faster than WT	2	10	35	1040	20%	3.4%	0.042

Supplementary Table 13: Enrichment of high-fitness variants based on language-model-recommended substitutions.

Each row corresponds to a protein tested via a high-throughput scanning mutagenesis assay that measures various notions of protein fitness, which are summarized in the "Fitness setting" column. All assays involve deep mutational scans that profile variants that

represent 90% or more coverage of all single-residue substitutions except for that of PafA, which changes every residue to either a glycine or a valine. The cutoff indicates the study-specific criterion for determining a high-fitness variant. The “Sample size” indicates the number of acquired variants ($|\mathcal{A}|$) and “Sample successes” indicates the number of those variants with high fitness according to the cutoff. The “Population size” indicates the number of variants profiled in the scanning mutagenesis assay, where “Population successes” indicates the number of those variants with high fitness according to the cutoff. “Hit rate” indicates the percentage fraction of high-fitness variants among the language-model-recommended variants (sample successes divided by sample size) whereas “Background” indicates the percentage fraction of high-fitness variants among all single-residue variants (population successes divided by population size). The hypergeometric P value computes enrichment of high-fitness variants among the acquired variants by assuming that the number of sample successes has a hypergeometric null distribution with parameters given by the other values (sample size, population successes, and population size); blue shading indicates a one-sided, hypergeometric P-value of less than 0.05.

Supplementary Information

Antibody sequences

Below are the antibody protein sequences defined as wildtype in this study:

- MEDI8852 VH:

  QVQLQQSGPGLVKPSQTLSLTCAISGDSVSSYNAVWNWIRQSPSRGLEWLGRTY
  YRSGWYNDYAESVKSRITINPDTSKNQFSLQLNSVTPEDTAVYYCARSGHITVFG
  VNVDAFDMWGQGTMVTVSS

- MEDI8852 VL:

  DIQMTQSPSSLSASVGDRVTITCRTSQSLSSYTHWYQQKPGKAPKLLIYAASSRGS
  GVPSRFSGSGSGTDFTLTISSLQPEDFATYYCQQSRTFGQGTKVEIK

- MEDI8852 UCA VH:

  QVQLQQSGPGLVKPSQTLSLTCAISGDSVSSNSAAWNWIRQSPSRGLEWLGRTY
  YRSKWYNDYAVSVKSRITINPDTSKNQFSLQLNSVTPEDTAVYYCARGGHITIFG
  VNIDAFDIWGQGTMVTVSS

- MEDI8852 UCA VL:

  DIQMTQSPSSLSASVGDRVTITCRASQSISSYLNWYQQKPGKAPKLLIYAASSLQS
  GVPSRFSGSGSGTDFTLTISSLQPEDFATYYCQQSRTFGQGTKVEIK

- mAb114 VH:

  EVQLVESGGGLIQPGGSLRLSCAASGFALRMYDMHWVRQTIDKRLEWVSAVGP
  SGDTYYADSVKGRFAVSRENAKNSLSLQMNSLTAGDTAIYYCVRSDRGVAGLF
  DSWGQGILVTVSS

- mAb114 VL:

  DIQMTQSPSSLSASVGDRITITCRASQAFDNYVAWYQQRPGKVPKLLISAASALH
  AGVPSRFSGSGSGTHFTLTISSLQPEDVATYYCQNYNSAPLTFGGGTKVEIK

- mAb114 UCA VH:

  EVQLVESGGGLVQPGGSLRLSCAASGFTFSSYDMHWVRQATGKGLEWVSAIGT
  AGDTYYPGSVKGRFTISRENAKNSLYLQMNSLRAGDTAVYYCVRSDRGVAGLF
  DSWGQGTLVTVSS

- mAb114 UCA VL:

  DIQMTQSPSSLSASVGDRVTITCRASQGISNYLAWYQQKPGKVPKLLIYAASTLQ
  SGVPSRFSGSGSGTDFTLTISSLQPEDVATYYCQKYNSAPLTFGGGTKVEIK

- S309 VH:

  QVQLVQSGAEVKKPGASVKVSCKASGYPFTSYGISWVRQAPGQGLEWMGWIST
  YNGNTNYAQKFQGRVTMTTDTSTTTGYMELRRLRSDDTAVYYCARDYTRGAW
  FGESLIGGFDNWGQGTLVTVSS

- S309 VL:

  EIVLTQSPGTLSLSPGERATLSCRASQTVSSTSLAWYQQKPGQAPRLLIYGASSRA
  TGIPDRFSGSGSGTDFTLTISRLEPEDFAVYYCQQHDTSLTFGGGTKVEIK

- REGN10987 VH:

  QVQLVESGGGVVQPGRSLRLSCAASGFTFSNYAMYWVRQAPGKGLEWVAVISY
  DGSNKYYADSVKGRFTISRDNSKNTLYLQMNSLRTEDTAVYYCASGSDYGDYL
  LVYWGQGTLVTVSS

- REGN10987 VL:

  QSALTQPASVSGSPGQSITISCTGTSSDVGGYNYVSWYQQHPGKAPKLMIYDVSK

  RPSGVSNRFSGSKSGNTASLTISGLQSEDEADYYCNSLTSISTWVFGGGTKLTVL

- C143 VH:

  EVQLVESGGGLVQPGGSLRLSCAASGFSVSTKYMTWVRQAPGKGLEWVSVLYS

  GGSDYYADSVKGRFTISRDNSKNALYLQMNSLRVEDTGVYYCARDSSEVRDHP

  GHPGRSVGAFDIWGQGTMVTVSS

- C143 VL:

  QSALTQPASVSGSPGQSITISCTGTSNDVGSYTLVSWYQQYPGKAPKLLIFEGTKR

  SSGISNRFSGSKSGNTASLTISGLQGEDEADYYCCSYAGASTFVFGGGTKLTVL
