# Supplementary material for: Efficient evolution of human antibodies from general protein language models
Source: Nat Biotechnol. 2023 Apr 24;42(2):275–83. doi: 10.1038/s41587-023-01763-2 (PMC10869273; doi:10.1038/s41587-023-01763-2)
Supplement: Supplementary file 2 — Reporting Summary [file 41587_2023_1763_MOESM2_ESM.pdf]

nature portfolio

Corresponding author(s): Brian L. Hie and Peter S. Kim

Last updated by author(s): Mar 13, 2023

Reporting Summary

Nature Portfolio wishes to improve the reproducibility of the work that we publish. This form provides structure for consistency and transparency in reporting. For further information on Nature Portfolio policies, see our Editorial Policies and the Editorial Policy Checklist.

Statistics

For all statistical analyses, confirm that the following items are present in the figure legend, table legend, main text, or Methods section.

n/a	Confirmed	
☐	☒	The exact sample size (*n*) for each experimental group/condition, given as a discrete number and unit of measurement
☐	☒	A statement on whether measurements were taken from distinct samples or whether the same sample was measured repeatedly
☐	☒	The statistical test(s) used AND whether they are one- or two-sided<br>*Only common tests should be described solely by name; describe more complex techniques in the Methods section.*
☐	☒	A description of all covariates tested
☐	☒	A description of any assumptions or corrections, such as tests of normality and adjustment for multiple comparisons
☐	☒	A full description of the statistical parameters including central tendency (e.g. means) or other basic estimates (e.g. regression coefficient) AND variation (e.g. standard deviation) or associated estimates of uncertainty (e.g. confidence intervals)
☐	☒	For null hypothesis testing, the test statistic (e.g. *F*, *t*, *r*) with confidence intervals, effect sizes, degrees of freedom and *P* value noted<br>*Give P values as exact values whenever suitable.*
☒	☐	For Bayesian analysis, information on the choice of priors and Markov chain Monte Carlo settings
☐	☒	For hierarchical and complex designs, identification of the appropriate level for tests and full reporting of outcomes
☐	☒	Estimates of effect sizes (e.g. Cohen's *d*, Pearson's *r*), indicating how they were calculated

Our web collection on statistics for biologists contains articles on many of the points above.

Software and code

Policy information about availability of computer code

Data collection	For data collection, we used the following commercial software applications: Octet System Data Analysis Software version 9.0.0.15 for biolayer interferometry data acquisition, PR.ThermControl version 2.3.1 for thermal melt data acquisition, BD CSampler Plus software version 1.0.34.1 for polyspecificity assay flow cytometry, and Tecan i-control version 2.0 for neutralization luminescence data acquisition. We used VWorks software version 13.1.0.1366 to control the Agilent Bravo robotic liquid handling platform.
Data analysis	Custom code for data analysis is available as Supplementary Code, at Zenodo at DOI:10.5281/zenodo.6977562, and at https://github.com/brianhie/efficient-evolution. We use the ESM-1b and ESM-1v language models from https://github.com/facebookresearch/esm. We used the Kabat region definition provided by the abYsis webtool version 3.4.1 (http://www.abysis.org/abysis/index.html) to annotate the framework regions and CDRs within the VH and VL sequences. We used NetMHCPan version 4.1 and NetMHCIIPan version 4.1 to predict peptide binders to class I and class II HLA. We perform data analysis with Python version 3.8; we also list individual package version information below. We used the neutcurve Python package version 0.5.7 to fit the normalized datapoints and to compute the IC50 values. We computed sequence similarity using the fuzzywuzzy Python package version 0.18.0. We used mafft version 7.475 to perform multiple sequence alignments.

For manuscripts utilizing custom algorithms or software that are central to the research but not yet described in published literature, software must be made available to editors and reviewers. We strongly encourage code deposition in a community repository (e.g. GitHub). See the Nature Portfolio guidelines for submitting code & software for further information.

Data

Policy information about availability of data

All manuscripts must include a data availability statement. This statement should provide the following information, where applicable:

- Accession codes, unique identifiers, or web links for publicly available datasets
- A description of any restrictions on data availability
- For clinical datasets or third party data, please ensure that the statement adheres to our policy

Raw data for this study has been deposited to Zenodo at DOI:10.5281/zenodo.6968342. Kd, IC50, and Tm values across replicate experiments are available as Supplementary Data 1. Median fluorescence intensity values for the polyspecificity experiments are available as Supplementary Data 2. Experimental values for our benchmarking of sequence-based methods and results from our UniRef90 parameter sweeps are available as Supplementary Data 3. High-likelihood amino acid substitutions for 742 therapeutic antibodies are available as Supplementary Data 4. Mean rank values for our DMS benchmark experiments are available as Supplementary Data 5. We also make use of the following publicly available databases and datasets:

• UniProt: https://www.uniprot.org/
• UniRef50 2018_03 [ref. 23]: https://ftp.uniprot.org/pub/databases/uniprot/previous_releases/release-2018_03/uniref/
• UniRef90 2020_03 [ref. 23]: https://ftp.uniprot.org/pub/databases/uniprot/previous_releases/release-2020_03/uniref/
• abYsis [ref. 45]: http://www.abysis.org/abysis/
• IMGT/LIGM-DB [ref. 70]: https://www.imgt.org/IMGTindex/LIGM-DB.php
• Thera-SAbDab [ref. 48]: https://opig.stats.ox.ac.uk/webapps/newsabdab/therasabdab/search/
• Livesey and Marsh benchmarking dataset [ref. 49]

Field-specific reporting

Please select the one below that is the best fit for your research. If you are not sure, read the appropriate sections before making your selection.

☒ Life sciences ☐ Behavioural & social sciences ☐ Ecological, evolutionary & environmental sciences

For a reference copy of the document with all sections, see nature.com/documents/nr-reporting-summary-flat.pdf

Life sciences study design

All studies must disclose on these points even when the disclosure is negative.

Sample size	To determine the number of antibody variants to screen, we use six language models to exhaustively search the complete space of single-residue substitutions to the antibody VH and VL sequences. Based on this exhaustive search, we chose an experimental sample size of ~10 for each round based on mutations with higher language model likelihood than wildtype across a consensus of two or more language models. A sample size of ~10 is a reasonable number of antibody variants for one person to express and purify in parallel using commonly used low-throughput antibody production techniques. Additional details are provided in Methods.<br><br>The number of variants measured in the scanning mutagenesis datasets were predetermined by previous studies, as described in references 8 and 72-79. All of these studies aimed to maximize mutational coverage of the positions in the protein sequence within the constraints of the respective high throughput technologies.
Data exclusions	No data were excluded from the analyses.
Replication	All attempts to replicate the data were successful. Biolayer interferometry data were obtained in duplicate or triplicate across multiple days and sample preparations, with the wildtype and highest-affinity samples also screened at multiple concentrations. Thermal melts were obtained across triplicate sample preparations. Polyspecificity data were obtained across three independent measurements. Neutralization data were obtained using duplicate wells replicated across two or more independent assays.
Randomization	Samples were allocated into groups independent of the experimental condition.
Blinding	Investigators were not blinded to experimental conditions. The language models recommended substitutions with no initial binding affinity data, knowledge of the antigen, task-specific supervision, evolutionary homologs, or protein structure information.

Reporting for specific materials, systems and methods

We require information from authors about some types of materials, experimental systems and methods used in many studies. Here, indicate whether each material, system or method listed is relevant to your study. If you are not sure if a list item applies to your research, read the appropriate section before selecting a response.

Materials & experimental systems

n/a	Involved in the study
☐	☒ Antibodies
☐	☒ Eukaryotic cell lines
☒	☐ Palaeontology and archaeology
☒	☐ Animals and other organisms
☒	☐ Human research participants
☒	☐ Clinical data
☒	☐ Dual use research of concern

Methods

n/a	Involved in the study
☒	☐ ChIP-seq
☒	☐ Flow cytometry
☒	☐ MRI-based neuroimaging

Antibodies

Antibodies used

Antibody wildtype sequences for the variable regions are provided in the Supplementary Information and new substitutions are listed in Supplementary Tables 2-9. Plasmid sequences are deposited at Zenodo at 10.5281/zenodo.6968342. Control antibody 4E10 was obtained from the HIV Reagent Program (ARP-10091). Control antibodies elotuzumab and ixekizumab were expressed and purified using the low-throughput methodology described in the paper. 0.001X goat anti-human Fab fragment FITC (Jackson ImmunoResearch, 109-097-003) was used to stain the polyspecificity assay.

Validation

All viral antibody plasmids were sequence confirmed across the full variable region. Antibodies were tested for protein expression via MabSelect purification, binding via biolayer interferometry, thermostability via thermal melting, and neutralization activity against infection with pseudotyped lentivirus; additional details are described in Methods. For polyspecificity assays, the values of the control antibodies were compared with known polyspecificity scores described in previous publications.

Eukaryotic cell lines

Policy information about cell lines

Cell line source(s)

Expi293F cells were obtained from ThermoFisher (catalog number A14527). HEK-293T cells were obtained from ATCC (catalog number CRL-3216). HeLa-ACE2-TMPRSS2 cells were obtained from the Jesse Bloom Laboratory (Fred Hutch).

Authentication

None of the cell lines were authenticated.

Mycoplasma contamination

All cell lines tested negative for mycoplasma contamination.

Commonly misidentified lines
(See ICLAC register)

None
